# Modular structure, sequence diversification and appropriate nomenclature of seroins produced in the silk glands of Lepidoptera

**DOI:** 10.1038/s41598-019-40401-3

**Published:** 2019-03-07

**Authors:** Lucie Kucerova, Michal Zurovec, Barbara Kludkiewicz, Miluse Hradilova, Hynek Strnad, Frantisek Sehnal

**Affiliations:** 1 Institute of Entomology, Biology Centre CAS, Branisovska 31, 370 05 Ceske, Budejovice, Czech Republic; 2 Faculty of Science, University of South Bohemia, Branisovska 31, 370 05 Ceske, Budejovice, Czech Republic; 3 Institute of Molecular Genetics CAS, Videnska 1083, 142 20, Prague, 4 Czech Republic

## Abstract

Seroins are small lepidopteran silk proteins known to possess antimicrobial activities. Several seroin paralogs and isoforms were identified in studied lepidopteran species and their classification required detailed phylogenetic analysis based on complete and verified cDNA sequences. We sequenced silk gland-specific cDNA libraries from ten species and identified 52 novel seroin cDNAs. The results of this targeted research, combined with data retrieved from available databases, form a dataset representing the major clades of Lepidoptera. The analysis of deduced seroin proteins distinguished three seroin classes (sn1-sn3), which are composed of modules: A (includes the signal peptide), B (rich in charged amino acids) and C (highly variable linker containing proline). The similarities within and between the classes were 31–50% and 22.5–25%, respectively. All species express one, and in exceptional cases two, genes per class, and alternative splicing further enhances seroin diversity. Seroins occur in long versions with the full set of modules (AB_1_C_1_B_2_C_2_B_3_) and/or in short versions that lack parts or the entire B and C modules. The classes and the modular structure of seroins probably evolved prior to the split between Trichoptera and Lepidoptera. The diversity of seroins is reflected in proposed nomenclature.

## Introduction

The silk spun by caterpillars is a composite material based on two protein agglomerates that have been known for centuries as fibroin and sericin. The fibroin assemblage creates the strong and elastic filaments, while sericin proteins are glues which seal the filaments (from the left and right silk gland) into a single fiber and provide fiber stickiness needed for the cocoon construction. A third kind of silk protein was discovered in the silk of the wax moth, *Galleria mellonella*, in 1998^1^. The proteins were called seroins to emphasize that they were secreted in the middle silk gland region like sericin proteins as well as in the posterior region like fibroin proteins. Two seroins found in the silk of *G. mellonella* proved to be glycoproteins of similar sizes (22.5 and 23 kDa) and with identical N-terminal sequences. A single cDNA, which was obtained with the aid of reverse transcription, encoded a peptide of 16 kDa. The cDNA sequence (GenBank Accession No. AF009828) was confirmed by Korayem *et al*.^2^, who showed that the gene was expressed not only in the silk gland, but also to a lesser extent in the midgut and fat body.

Two similar proteins of 8 and 13 kDa were detected by N-terminal microsequencing in *Bombyx mori* silk and the cDNAs encoding these proteins were isolated from the silk gland mRNAs^3^. The deduced peptides, which were designated Seroin 1 (predicted size 9.9 kDa, GenBank No. NM_001043580) and Seroin 2 (10.3 kDa, NM_001043581) differed from one another, but the spacing of some amino acid residues was similar to that in the seroin of *G. mellonella*. It was tentatively concluded that *G. mellonella* contains a single *seroin* gene, while *B. mori* harbours two *seroin* paralogs. These suggestions were confirmed by other researchers who also demonstrated differences in the expression of the two *Bombyx* seroins in different tissues^4–6^.

*Seroin*-like transcripts were also found in moths of the Saturniidae family. Gandhe *et al*.^7^ detected one (GenBank No. DQ666525), and Maity *et al*.^8^ distinguished two seroin orthologs expressed in the integument, fat body, midgut and silk glands of *Antheraea mylitta* larvae. The deduced proteins had identical N-terminal sequences, suggesting that they might be derived from a single alternatively spliced gene. Tsubota *et al*.^9^ prepared two cDNA libraries for *Samia cynthia ricini*: one specific for the anterior + middle silk gland regions (A/MSG) and the other for the posterior region (PSG). Four contigs regarded as putative seroin genes (LC001871–LC001874) were found. Two of them were abundant in the A/MSG library and two in the PSG library. Singh *et al*.^5^ used Seroin 1 and Seroin 2 of *B. mori* as queries to screen cDNA sequences deposited in GenBank (NCBI), and found nine new seroins in eight lepidopteran species. They classified all these novel seroins as orthologs of *B. mori* Seroin 2, while Seroin 1 was detected only in *B. mori* and its wild relative *Bombyx huttoni*. The authors suggested that *B. mori* Seroin 2 is more related to the original lepidopteran seroin gene that was duplicated in ancestors of the *Bombyx* genus. Dong *et al*.^6^ also searched for seroin homologs in the NCBI non-redundant protein database and found 64 seroins in 32 lepidopteran species. Based on these data they concluded that there are on average two seroin paralogs in each species. However, incomplete amino acid sequences were often used to recognize seroins, and their assessment of the relationships and possible role in differential gene splicing in seroin diversity received little attention. Therefore, earlier suggested seroin domain structure and classification require revision and complementation, which is the aim of this paper. Data on the distantly related species and complete and verified cDNA sequences are used to build a reliable knowledge base of seroins. The distinction of orthologs and paralogs is essential for the accurate interpretation of the evolutionary relationships of seroins as well as for comparative and functional studies, including elucidation of the role of seroin in silk and other secretions of the labial glands. We show that lepidopteran species usually contain three seroin paralogs. This report contributes to seroin cognizance by addressing seroin classification, nomenclature, modular structure, splicing, diversity and phylogeny.

## Results and Discussion

### Seroin classification

Dong *et al*.^6^ analyzed 64 seroins from 32 species and, on the basis of conserved C-terminal regions (50–54 amino acid residues), recognized five seroin subfamilies composed of three kinds of structural domains. Every domain allegedly included a proline-rich N-terminal region and the more conserved C-terminal region. The seroins of *B. mori* were taken as prototypes of domains 1 and 2, and the remaining sequences were assembled in a separate domain 3. Seroin subfamilies 1, 2 and 3 contained single domains 1, 2 and 3, respectively, while domain combinations 2 + 1 and 3 + 3 comprised the subfamilies referred to with the same designations 2 + 1 and 3 + 3. We attempted to use this system of domains and subfamilies for seroin classification but we found it unsuitable because it was not based on phylogenetic relationships, did not take into account alternative splicing, and ignored the N-terminal regions of the domains.

**Figure 1:**
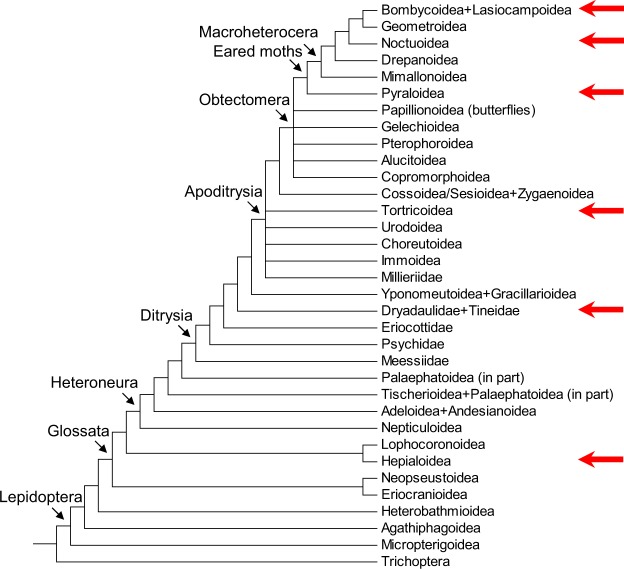
Simplified cladogram of lepidopteran superfamilies. The superfamilies represented in our RNA sequencing analysis are indicated by arrows on the right side. Based on Mitter *et al*.^15^ and Regier *et al*.^23^.

We conceived seroin classification based on the full-length cDNAs detected in the silk gland specific transcriptomes of species representing the major lepidopteran clades (Fig. 1). Additional cDNAs encoding seroins were retrieved from public sources. In total we examined 117 different seroin cDNAs from 33 lepidopteran species (Table 1). Tentative alignments of the encoded proteins performed by MUSCLE algorithm^10^ indicated that seroins expressed in the silk glands of Lepidoptera belonged to three classes denoted Sn1, Sn2 and Sn3. The classes were defined by the spacing and modular arrangement of short amino acid motifs. The Sn1 class, represented by 61 cDNAs, was characterized by the high content of charged amino acid residues in the vicinity of Trp located ca 20 residues after the initial Met, and by a distinct Pro-rich region that followed afterwards (Fig. S1). The Sn2 class (33 cDNAs) was distinguished by the relatively high content of Gly within the first 20–50 residues and by the RYGG motif found in 17 seroins of this class (Fig. S2). Out of 23 Sn3 proteins, 18 contained Trp located ca 35 residues from the initial Met, of which 17 contained a VYGE motif that occurs only in Sn3 (Fig. S3).

**Table 1:** Affiliation of analyzed Lepidopteran species to families and superfamilies.

Species name and abbreviation	Superfamily	Family	Data origin*	Numbers of seroins in classes**		
				Sn1	Sn2	Sn3
*Hepialus californicus* Hc	Hepialoidea	Hepialidae	Illumina	2	1	?
*Tineola bisselliella* Tb	Tineoidea	Tineidae	Illumina	1	?	1
*Telchin licus* Tl	Sesioidea	Castniidae	(GenBank data)	1	?	?
*Cydia pomonella* Cp	Tortricoidea	Tortricidae	Roche 454	4	?	1
*Choristoneura fumiferana* Cf	Tortricoidea	Tortricidae	(GenBank data)	2	?	?
*Grapholita molesta* Gmo	Tortricoidea	Tortricidae	(GenBank data)	1	?	?
*Carposina sasakii* Cs	Carposinoidea	Carposinidae	(GenBank data)	1	?	?
*Ostrinia nubilalis* On	Pyraloidea	Crambidae	Illumina	4	2	1
*Ostrinia furnacalis* Of	Pyraloidea	Crambidae	(GenBank data)	2	?	?
*Plodia interpunctella* Pi	Pyraloidea	Pyralidae	Lepbase	1	1	1
*Amyelois transitella* At	Pyraloidea	Pyralidae	(GenBank data)	4	1	2
*Anagasta kuehniella* Ak	Pyraloidea	Pyralidae	Roche 454	4	1	?
*Galleria mellonella* Gm	Pyraloidea	Pyralidae	Roche 454	4	3	1
*Operophtera brumata* Ob	Geometroidea	Geometridae	(GenBank data)	?	?	1
*Acanthobrahmea europea* Ae	Bombycoidea	Brahmeidae	Illumina	2	3	1
*Samia ricini* Sr	Bombycoidea	Saturniidae	(GenBank data)	3	?	?
*Antheraea yamamai* Ay	Bombycoidea	Saturniidae	Roche 454	3	2	1
*Antheraea mylitta* Am	Bombycoidea	Saturniidae	(GenBank data)	1	?	?
*Bombyx mori* Bm	Bombycoidea	Bombycidae	(GenBank data)	2	3	1
*Bombyx huttoni* Bh	Bombycoidea	Bombycidae	(GenBank data)	2	?	?
*Spodoptera exigua* Se	Noctuoidea	Noctuidae	(GenBank data)	?	?	2
*Spodoptera litura* Sl	Noctuoidea	Noctuidae	(GenBank data)	1	2	1
*Spodoptera littoralis* Sli	Noctuoidea	Noctuidae	Illumina/GenBank	3	2	1
*Mamestra brassicae* Mb	Noctuoidea	Noctuidae	Illumina	3	2	?
*Helicoverpa armigera* Ha	Noctuoidea	Noctuidae	(GenBank data)	1	2	1
*Helicoverpa assulta* Has	Noctuoidea	Noctuidae	(GenBank data)	?	?	1
*Agrotis segetum* As	Noctuoidea	Noctuidae	(GenBank data)	?	?	1
*Papilio xuthus* Px	Papilionoidea	Papilionidae	(GenBank data)	2	1	1
*Papilio machaon* Pm	Papilionoidea	Papilionidae	(GenBank data)	2	1	1
*Papilio polytes* Pp	Papilionoidea	Papilionidae	(GenBank data)	1	4	1
*Pieris napi* Pn	Papilionoidea	Pieridae	Lepbase	3^+^	1	1
*Danaus plexipus* Dp	Papilionoidea	Nymphalidae	(GenBank data)	?	1	1
*Heliconius melpomene* Hm	Papilionoidea	Nymphalidae	Lepbase	1	?	?

The source of data and the numbers of cDNA forms of indicated seroin classes (listed from the ancestral to the derived superfamilies).

*New data were obtained by transcriptome sequencing using the pyrosequencing on Roche 454 or the Illumina method. Additional data were drawn from the GenBank, Lepbase or provided by the colleagues listed in the Acknowledgements.

**Numbers of seroins in each class. Questionmarks indicate that the absence of a class could be double checked by additional data screening or independent reverse transcription.

^+^Number of transcripts derived from the Seroin 1 gene in *P. napi* (PnSn1) is uncertain and is expected to be higher.

The alignment of complete amino acid sequences proved to be an easy and reliable way to distinguish seroin classes Sn1, Sn2 and Sn3, which represent three different types of phylogenetically related seroin genes. The alignments also revealed that these classes only partially overlapped with the seroin domains described by Dong *et al*.^6^, since they did not distinguish variability caused by gene relationship and alternative splicing. These authors proposed the Seroin 1 and Seroin 2 of *B. mori*^3^ to be used as prototypes of the domains 1 and 2. However, our data clearly show that the pattern of seroin sequences in *Bombyx* represents a derived trait. These silkworm seroins, and also other seroins these authors listed as domain 2, align within the class Sn1 (Fig. S4). In addition, all seroins reported as containing domains 2 + 1^11^ are long transcriptional variants of class Sn1 and the seroins of domains 3 and 3 + 3 are the short and long versions of the Sn3 class, respectively (see section “Long and short seroin versions”). The Sn2 class represents an entirely novel group that contains new kinds of seroins. Our alignment of full cDNAs sequences in most cases allows distinction between transcripts encoded by separate genes and transcripts derived from a single gene via differential splicing. The alignments demonstrate the presence of conserved amino acids and reveal similar structural patterns. Similarity within classes reaches 31–50% and between classes 22.5–25%.

### Seroin nomenclature

The new classification of seroins based on the full-length cDNAs encouraged us to propose a nomenclature that reflects seroin diversity, specifies the seroin genes and differentiates between the products of different genes and the splicing variants of a single gene. The name of each seroin starts with the abbreviation of insect genus and species, for example Bm for *Bombyx mori* (we use a two-letter code but it may be longer if needed, e.g. as for *Helicoverpa assulta* to avoid confusion with Ha, *Helicoverpa armigera*), followed by the class designation Sn1, Sn2 or Sn3. While the available transcriptomic and genomic data suggest that each species contains a single gene of each seroin class, both the Sn1-specific pattern of transcripts of *B. mori* and the genomic sequences of *B. mori* and *B. mandarina* (http://silkbase.ab.a.u-tokyo.ac.jp/cgi-bin/news.cgi) show that there are two paralogs of Sn1 in both Bombyx species. In such instances, an extended gene name containing a hyphen and the serial gene number 1 or 2 (more than 2 genes of a class were never detected) are inserted into the seroin name after class designation; for example, BmSn1-1 and BmSn1–2 (Seroin 1 and Seroin 2 according to Nirmala *et al*.^3^) specify two *B. mori* paralogous genes of class Sn1. No extended gene numbering is necessary in Lepidoptera that contain a single gene per class and the gene is therefore unequivocally identified by insect species and seroin class. The splicing versions are distinguished by capital letters inserted after the gene specification. Hence, splicing versions of the only GmSn1 gene of *Galleria mellonella* are designated GmSn1A, GmSn1B and GmSn1C. An additional number after the splicing variant letter at the end of the name indicates a minor sequence difference (very short exons, etc.).

### Long and short seroin versions

Proteins containing identical N-termini are often products of an alternative splicing of a single gene. However, unless we elucidate the genomic sequence, we cannot exclude that even very similar cDNAs are derived from different genes. For example, several closely related Sn1 transcripts were detected in *B. mori* and *Pieris napi* but only an analysis of the genomic sequences^12,13^ revealed that all *P. napi* transcripts are products of a single gene, whereas two short transcripts found in the silkworm are products of two separate genes. In *Pieris*, the sequences of multiple short ESTs are contained within the full length of the Sn1 transcript, while in *Bombyx* there are only two Sn1-like transcripts with identical 5′ ends; the remaining regions of the transcripts reflect differences between the two paralog Sn1 genes of *B. mori*. Most Sn1 occur in multiple size isoforms with overlapping sequences, strongly suggesting that they are generated by alternative splicing.

In *G. mellonella* we found one long Sn1 version (L) covering the entire ORF, and shorter N-terminal and C-terminal versions (Fig. S1). All deduced proteins contain identical N-termini and the longest isoform includes all shorter variants, suggesting that the cDNAs are derived from the single gene *GmSn1* (Table S1). Similar modes of transcript splicing occur also in *C. pomonella* and *O. nubilalis*. The comparison of deduced proteins shows that each of the shorter cDNAs often encodes about one half of the full-length seroin protein (Table S1). The alignment of 61 Sn1 sequences clearly distinguishes between the L-isoforms and the shorter N and C isoforms (Fig. S1) that are represented in our Sn1 set by 18, 17 and 26 specimens, respectively (Table 2). Most species seem to splice the transcript preferably or exclusively in one way that generates a specific version(s) of Sn1. For example, *A. transitella* and *A. kuehniella* each produce several closely related long Sn1 versions but only one C-terminal short version was found and only in *A. kuehniella*.

**Table 2:** Classification of identified seroins and recognition of conserved splicing versions in class Sn1.

Species	Splicing versions of Class 1 seroins			Class 2	Class 3
	N-terminal	C-terminal	Long		
*Hepialus californicus*	—	—	HcSn1A, HcSn1A2	HcSn2	?
*Tineola bisselliella*	TbSn1	—	—	?	TbSn3
*Telchin licus*	?	?	TlSn1	?	?
*Cydia pomonella*	CpSn1A	CpSn1B,CpSn1B2	CpSn1C	?	CpSn3
*Choristoneura fumiferana*	?	CfSn1B	CfSn1A	?	?
*Grapholita molesta*	GmoSn1	?	?	?	?
*Carposina sasakii*	?	?	CsSn1	?	?
*Ostrinia nubilalis*	OnSn1A, Osn1A2	OnSn1B	OnSn1C	OnSn2A, OnSn2B	OnSn3
*Ostrinia furnacalis*	?	OfSn1B	OfSn1A	?	?
*Plodia interpunctella*	PiSn1	?	?	PiSn2	PiSn3
*Amyelois transitella*	—	—	AtSn1A, AtSn1B, AtSn1C, AtSn1D	AtSn2	AtSn3A, AtSn3B
*Anagasta kuehniella*	—	AkSn1A	AkSn1B, AkSn1B2, AkSn1C	AkSn2	?
*Galleria mellonella*	GmSn1A	GmSn1B, GmSn1B2	GmSn1C	GmSn2A, GmSn2B, GmSn2C	GmSn3
*Operophtera brumata*	?	?	?	?	ObSn3
*Acanthobrahmea europea*	AeSn1A	AeSn1B	—	AeSn2A, AeSn2B, AeSn2C	AeSn3
*Samia ricini*	SrSn1A, SrSn1C	SrSn1B	—	?	?
*Antheraea yamamai*	AySn1A, AySn1C	AySn1B	—	AySn2A, AySn2B	AySn3
*Antheraea mylitta*	AmSn1	?	?	?	?
*Bombyx mori*	BmSn1–2	BmSn1-1	—	BmSn2A, BmSn2B, BmSn2C	BmSn3
*Bombyx huttoni*	BhSn1–2	BhSn1-1	—	?	?
*Spodoptera exigua*	?	?	?	?	SeSn3A, SeSn3A2
*Spodoptera litura*	?	?	SlSn1	SlSn2A, SlSn2B	SlSn3
*Spodoptera littoralis*	SliSn1A2, SliSn1A4	SliSn1A	—	SliSn2A, SliSn2B	SliSn3
*Mamestra brassicae*	MbSn1B	MbSn1A, MbSn1A2	—	MbSn2A, MbSn2B	?
*Helicoverpa armigera*	?	HaSn1	?	HaSn2A, HaSn2B	HaSn3
*Helicoverpa assulta*	?	?	?	?	HasSn3
*Agrotis segetum*	?	?	?	?	AsSn3
*Papilio xuthus*	?	?	PxSn1A, PxSn1B	PxSn2	PxSn3
*Papilio machaon*	?	?	PmSn1A, PmSn1B	PmSn2	PmSn3
*Papilio polytes*	?	?	PpSn1	PpSn2A, PpSn2B, PpSn2C, PpSn2D	PpSn3
*Pieris napi*	?	?	PnSn1A, PnSn1B, PnSn1C	PnSn2	PnSn3
*Danaus plexipus*	?	?	?	DpSn2	DpSn3
*Heliconius melpomene*	?	?	HmSn1	?	?

*Seroins are unequivocally identified with the newly proposed nomenclature (section seroin nomenclature). Question marks show that available data might be insufficient for detection of the respective seroin.

**Figure 2:**
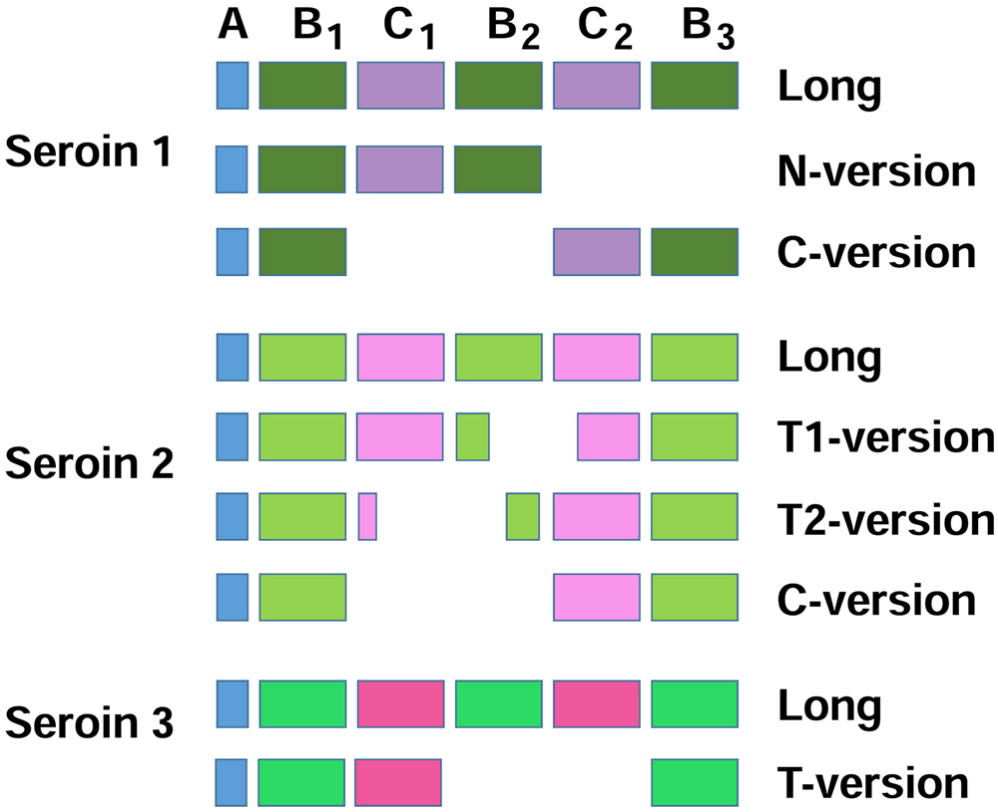
Comparison of the domain organization and major splice isoforms of Sn1, Sn2 and Sn3 proteins. Seroins occur in long versions with the full set of modules (AB_1_C_1_B_2_C_2_B_3_) and/or in short versions that lack parts or the entire B and C modules. The modules are indicated by color boxes. Three major splicing isoforms are generated in Sn1, four isoforms in Sn2 and two isoforms in Sn3. The alignment depicts the presence of individual modules in different isoforms.

The finding of different Sn2 isoforms in 10 out of 16 species confirms that alternative splicing is an important mechanism of seroin diversification (Fig. S2). The production of the Sn2 L-version and C-version resemble the situation in Sn1 but the patterns of splicing are different. In contrast to Sn1, all Sn2 transcripts retain at least part of the Gly-rich sequence following after the signal peptide (30–40 a.a.). Sn2 are also uniquely spliced to two versions that contain both N-terminal and C-terminal regions but have lost large blocks from the internal sequence. These versions are called terminal T1 and T2 (Fig. 2).

The Sn3 seroins occurred in two isoforms that differed in size. We found short Sn3 versions (120–170 amino acid residues) in 15 different species (Fig. S3), whereas Dong *et al*.^11^ detected exclusively long Sn3 versions (which they referred to as the 3 + 3 subfamilies) in all five noctuids they examined. The N-terminal, as well as the C-terminal regions of Sn3, are identical in both long and short isoforms but the central region of about 140 residues is present only in the L-versions (Fig. S3). We were able to confirm the presence of long Sn3 in the *Spodoptera frugiperda* v2 assembly^14^, which is available in Lepbase^12^. Available evidence suggests that the long Sn3 versions are specific for Noctuoidea but we failed to detect the long Sn3 versions in our transcriptome data from the two noctuid species (*M. brassicae* and *S. littoralis*) that we analyzed, although we already had the long Sn3 sequence of *S. littoralis* available from GenBank (EZ983678). This may have been caused by a very low expression of the Sn3 seroins. By comparing the numbers of class-specific reads in several transcriptomes, we found that the level of expression of Sn2 and Sn3 is much lower than Sn1 (Table 3).

**Table 3:** Comparison of the number of reads recognized as class specific in several silk gland transcriptomes.

Sample (cDNA)	Total number of reads for the library	Sn1 reads	Sn2 reads	Sn3 reads
*G. mellonella* Penn inst	68365	1083	97	3
*G*. *mellonella* Wander. st.	76492	3428	154	12
*G. mellonella* Prepupa	81015	4954	300	40
*A. yamamai*	120793	6919	6	701
*C. pomonella*	76434	214	0	8
*A. kuehniella*	76439	346	1	0
*O. nubilalis*	25429	60	1	1
*A. europaea*	32551	6	3	1

*TBLASTN search was used to identify the reads; query sequence: 80 C-terminal amino acids, threshold e-value was set to e^−20^; Penn inst -penultimate instar larvae; Wander st. - post-feeding wandering last-instar larvae, Prepupa - apolyzing (initial phase of pupation) last-instar larvae.

### The modular structure of seroins

Nascent seroin proteins contain signal peptides of ca 20 amino acid residues (module A in Fig. 2). Mature proteins are up to 250 amino acid residues long and are composed of structural modules. The modules are not sharply defined but are easily recognized in the seroin alignments (Figs S1–S3). The conserved blocks of amino acid motifs containing a high proportion of Ala, Ser, Glu and Asp are referred to as B modules and the shorter, diversified and mostly proline-rich regions are called the C modules. The modules are numbered consecutively within each seroin class. Some seroins of all classes occur in long versions composed of the full sets of modules AB_1_C_1_B_2_C_2_B_3_ and in shorter versions that are more class-specific: the N-terminal version AB_1_C_1_B_2_ occurs in Sn1, the C-terminal version AB_1_C_2_B_3_ in Sn1 and Sn2, the terminal T1 and T2 versions (AB_1_C_1_[B_2_C_2_]B_3_ and A[B_1_C_1_]B_2_C_2_B_3_) in Sn2 (modules in brackets are incomplete) and the T-version AB_1_C_1_B_3_ in Sn3 (Fig. 2). The similarities of seroins in diverse Lepidoptera indicate that the modular structure evolved in ancient Lepidoptera and has remained relatively conserved. In comparison with other seroin classes, the B_1_ module of Sn1 is short, C_1_ is well developed and very proline-rich, B_2_ is large, C_2_ is diversified and reduced, and B_3_ is somewhat larger than B_2_ (Fig. S1). The long seroin isoforms of class 2 (Fig. S2) include a relatively glycine-rich B_1_ of ca 30–50 residues, a short putative C_1_, a B_2_ that is variable in length (25–50 residues), a short putative C_2_ (ca 30 residues), and a large B_3_ (over 90 residues). The Pro content is usually increased in both C_1_ and C_2_ of Sn2 (except for AySn2B or GmSn2C where only one Pro appears in C1). Distinct long and short seroin versions occur in Sn3 (Fig. S3). Both versions include the modules B_1_ (ca 40 residues), C_1_ (10–35 residues) and B_3_ but the internal modules B_2_C_2_ are present only in the long version of Sn3.

### Seroin diversity within Lepidoptera

The silk gland transcriptomes of Lepidoptera typically harbor three to six kinds of seroin cDNAs (most of them are splicing isoforms of Sn1); lower numbers were mostly reported in the insufficiently examined species and are probably underestimated (Table 1). From the silk gland specific EST library of *Hepialus californicus* (Hepialoidea), we retrieved two long cDNAs tentatively classified as Sn1 but hardly reduced C_2_ module (Fig. S1). Another cDNA resembled the Sn2 class but contained a unique B_1_ and lacked the C_1_ and about half of the B_2_ modules (Fig. S2). Two kinds of seroin cDNAs were also identified in *Tineola bisselliella* (Tineoidea). One kind encoded a short N-terminal version of Sn1 but had a very short B_1_ module and therefore exhibited only low sequence similarity with the Sn1 peptides of other Lepidoptera (Fig. S1). The other cDNA of *T. bisselliella* aligned well with the Sn3 sequences of the evolutionarily more advanced moths. The data available for Hepialoidea and Tineoidea suggest that the modular structure of three seroin classes and the seroin genes splicing to the long and short mRNA versions appeared in the early evolution of Lepidoptera and became stabilized in the clade Apoditrysia (Fig. 1).

The transcriptome of *Cydia pomonella* from Tortricoidea included cDNAs encoding two short and one long versions of CpSn1 (Fig. S1). A similar splicing pattern of Sn1 can be deduced from the data published by Dong *et al*.^6^ for the related tortricoid species *Choristoneura fumiferana*. All examined Pyraloidea also contained differentially spliced genes that yielded several isoforms of Sn1 proteins (Table S1). The simultaneous production of one long and two short splicing isoforms of Sn1 was found in *Galleria mellonella* and in *Ostrinia nubilalis*. In *Anagasta kuehniella* we detected three long and one short C-terminal isoform of Sn1. The genomic sequence of *Amyelois transitella* contained four differentially spliced long cDNAs (Table 2). However, typical pyraloids express only one differentially spliced gene of each class (Table 1).

The Sn1 cDNAs found in Bombycoidea exclusively encode the shorter versions of seroin proteins. *Acanthobrahmaea europea* contains one N-terminal and one C-terminal Sn1 isoform and *Antheraea yamamai* harbors two N-terminal and one C-terminal Sn1. Both species express a single Sn1 gene that is alternatively spliced. The length of encoded peptides is reduced due to stop codons present in the 3´ region of the transcripts. *B. mori*, *B. huttoni* and most probably *Samia ricini*, contain two paralogous *Sn1* genes, one encoding a cDNA analogous to the N-terminal and the other analogous to the C-terminal region of the long Sn1 transcripts (Table S1). Seroins Sn2 and Sn3 are each represented in Bombycoidea by one gene per class; class Sn2 occurs in two splicing versions in AySn2 and in three versions in AeSn2 and BmSn2. Sn2 transcripts exist in four splicing isoforms: (1) long transcripts including the entire ORF (e.g. BmSn2A, AySn2A, AeSn2A), (2) slightly shortened versions with missing parts of the B_1_ and C_1_ modules (BmSn2C, AySn2B), (3) missing parts of the B_2_ and C_2_ domains (BmSn2B, AeSn2B) or an extra short transcript, with a missing C-terminal part from half of B_1_, over the whole C_1_ and B_2_ (AeSn2C). For Sn3 in Bombycoidea we identified only a single transcription variant.

The silk gland transcriptomes analyzed in our study included Noctuoidea *Spodoptera littoralis* and *Mamestra brassicae*, in which we found only the short splicing variants of Sn1 (both N-terminal and C-terminal, Fig. S1). By contrast, a long Sn1 cDNA found in *Spodoptera litura* was available in GenBank (XM 022965570). Within the Sn2 class, we consistently identified one long transcript (MbSn2A, SlSn2A, SfSn2A, HaSn2A) and one slightly shorter transcript with missing parts of the B_2_ and C_2_ modules (MbSn2B, SlSn2B, SfSn2B, HaSn2B) (Fig. S2). The sequences of Sn3 in *Spodoptera exigua*, *S. littoralis*, *S. litura*, *Helicoverpa assulta*, *H. armigera* and *Agrotis segetum* were retrieved from GenBank. All of them contained long N-terminal and C-terminal regions, each of ca 60 residues, matching the Sn3 ends in other Lepidoptera (Fig. S3). The central part of the sequence of ca 130 residues was present only in Sn3 of the Noctuid species listed above. Surprisingly, no long versions of Sn3 were detected in our transcriptomes based on the silk glands of *S. littoralis* and *M. brassicae* (see section Long and short seroin versions).

Our data on the superfamily Papilionoidea are based upon information retrieved from Lepbase^12^. The long version of Sn1 was detected in *Heliconius melpomene* and in the *Papilio* species *P. xuthus*, *P. machaon* and *P. polytes* (Fig. S1). The presence of one short C-terminal Sn1 transcript was predicted for *P. napi* based on the partial genomic sequence but the work has not been completed. Several seroins represent unique splicing isoforms: PxSn1B is extended by a duplication of 118 residues at the C terminus, PmSn1B contains an insertion of ca 60 residues between modules B_2_ and B_3,_ and PnSn1C lacks the B_1_ module and part of the C_1_ module. *Papilio* species, *D. plexipus* and *P. napi* also contain Sn2 seroins (Fig. S1); several transcription variants of Sn2 were found in *P. polytes*. The orthologs of Sn3 were found in the *Papilio* species, *P. napi* and in *Danaus plexipus* (Fig. S3).

### Seroin evolution

**Figure 3:**
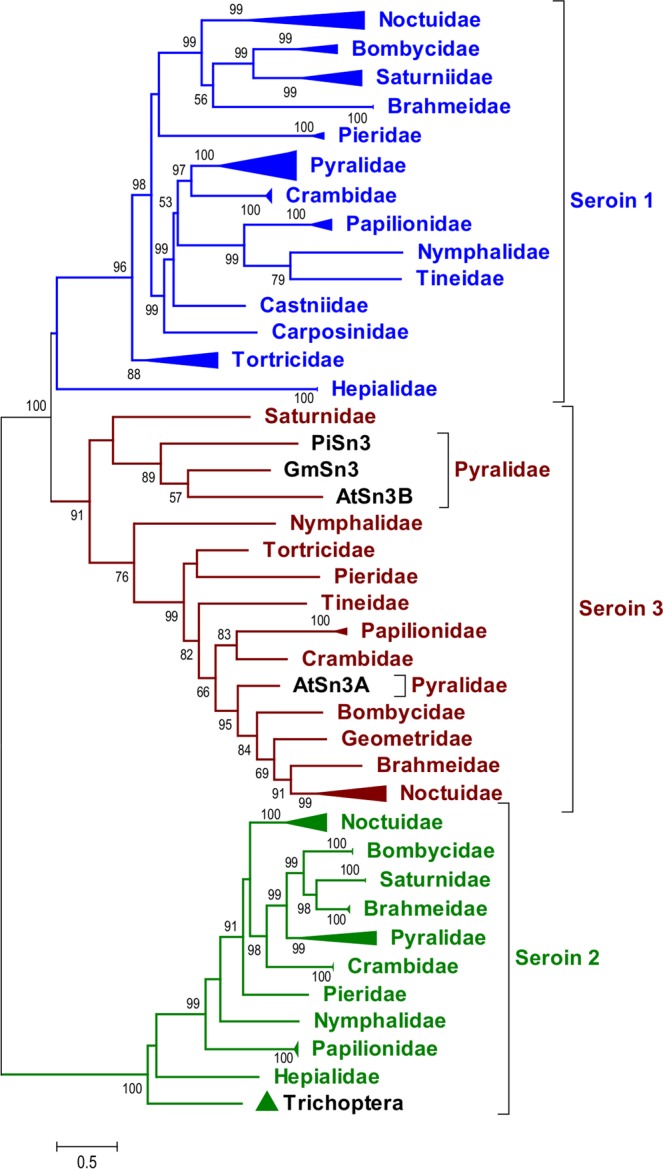
Simplified family-level phylogeny of seroins. The full-size ML phylogenetic tree is shown in Fig. S5A. The broadenings of horizontal lines indicates inclusion of more than one species in the respective family. Statistical evaluation calculated by aBayes test is shown next to the branches, only values higher than 50 are presented.

In order to understand the evolutionary history of the seroin gene family we used several methods to construct sequence alignments and phylogenetic trees of the deduced seroin proteins. The entire translated ORF was used for comparison. Because of splice variant complexity, we used only character-based methods. We obtained phylograms with similar topology in the Maximum likelihood trees (Fig. S5A,B) and in the Bayesian inference tree (Fig. S5C). The data strongly support seroin division into three classes (aBayes values between 90–100 and posterior probabilities between 0,96–1 were obtained in all cases) that are represented by deep branches in the phylogenetic trees. We assume that the formation of classes was driven by successive gene duplications, which is also supported by gene localization within close proximity, e.g. all three seroin genes from *P. napi* are localized on chromosome 15 close to each other^12^, seroin genes are localized next to each other also in *B.mori* chromosome 5 (http://silkbase.ab.a.u-tokyo.ac.jp/cgi-bin/news.cgi). An indigenous seroin gene probably duplicated and thereby yielded Sn2 and the joint precursor of Sn1 and Sn3. We speculate that Sn2 seceded from the ancestral seroin stock very early in the evolution of Lepidoptera, possibly already in their predecessors shared with the Trichoptera. The Sn2 separation from the precursor of the other two seroin branches is maximally supported by all phylograms (aBayes value 100, posterior probabilities 1). The Sn2 branch includes seroins from advanced Ditrysia but no Sn2 was detected in the less advanced Ditrysia represented by the superorders Tineoidea and Tortricoidea. On the other hand, Sn2-like homologs were found in the primitive moth *Hepialus californicus* and in the Trichoptera species *Oligotricha striata* (Fig. 3). These seroins are probably close to the ancestral Sn2.

Sn1 and Sn3 split from one another more recently. The Sn1/Sn3 duplication may have occurred after the separation of Hepialoidae from the stock of other primitive superfamilies. This assumption is based on the apparent absence of the Sn3 gene and the presence of a prototypical Sn1 gene in *H. californicus*. The positions of the HcSn1A and HcSn1A2 branches are not clear. Bayesian inference (BI) analysis placed them in a trichotomy with the Ditrysian Sn1 and Sn3 branches (Fig. S5C), while the Maximum-Likelihood (ML) method grouped them with the Sn1 or Sn3 sequences at aBayes values below 50% (Fig. S5A,B), 0–33% respectively.

After the initial split of seroins into three classes, each class apparently diversified independently. Close structural similarity has been maintained at the level of families within each superfamily (Fig. 3). The patterns of seroin clusters reflect the evolutionary relationships of Lepidoptera at different taxonomic levels. Each species typically harbors one Sn gene per class. Two Sn1 paralogs occur in *B. mori* and most probably also *B. huttoni*. The complexity of seroins in modern species suggests considerable diversification and in some cases also gene duplication. Numerous seroin isoforms are produced by alternative splicing. The newly proposed seroin nomenclature defines insect species and seroin class and specifies the gene and its splicing isoforms.

## Materials and Methods

### The choice of insects

Lepidoptera is a large and diversified insect order with nearly 200,000 species assigned to 46 superfamilies^15^. Several primitive superfamilies are clustered in the polyphyletic clade Monotrysia that was represented in our study by the superfamily Hepialoidea (Fig. 1). About 98% of Lepidoptera belong to the monophyletic Ditrysia that harbor a few basal superfamilies with uncertain relationships; we chose a representative of Tineidae. Ditrysia further include the well-defined and obviously monophyletic clades Apoditrysia, Obtectomorpha and Macroheterocera^15^. The superfamilies used in our research represent all of these clades. (Fig. 1) We prepared silk gland cDNA libraries from ten species for transcriptome sequencing and exploited data available in Lepbase^12^ and GenBank on 23 additional species. We have also prepared an RNA-Seq Library from *Oligotricha striata* representing the Trichoptera (a sister order of Lepidoptera). All analyzed Lepidopteran species are listed in alphabetic order in Table 1.

### Transcriptome preparation

The purification of total RNA from the silk glands, cDNA preparation, and pyrosequencing were employed in four species (*Galleria mellonella*, *Anagasta kuehniella*, *Cydia pomonella* and *Antheraea yamamai*, Table 1) as described in Zurovec *et al*.^16^. The silk gland RNA samples from six other species (*Tineola bisselliella*, *Ostrinia nubilalis*, *Mamestra brassicae*, *Acanthobrahmaea europaea*, *Hepialus californicus*, *Spodoptera littoralis*) were used to prepare cDNA libraries for the Illumina sequencing platform. A RiboMinus Eukaryote Kit for RNA-Seq (Ambion) was used to remove rRNA, the poly-A mRNA was enriched with the aid of a Dynabeads Oligo (dT)25 mRNA Purification Kit (ThermoFisher Scientific), and the cDNA library was created with a NEXTflex Rapid RNA-Seq Kit (Bioo Scientific). The sequencing was performed with an MiSeq (Illumina) instrument producing sequences in the 2 × 250 nt pair-end format. The sequences were trimmed using Trimmomatic software (v. 0.32)^17^ with the quality scores of Q19 and minimum length 40 bp. The *de novo* transcriptome assembly was performed using Trinity software (v. 2.1.1.) using the default settings. The resulting transcriptome of *Tineola bisselliella* contained 1830404 reads and the assembly predicted 19024 ‘genes’ (all transcript contigs N50: 714 bp); *Ostrinia nubilalis* contained 1890578 reads and 17253 predicted ‘genes’ (N50: 525 bp); *Mamestra brassicae* contained 1705429 reads and 18579 ‘genes’ (N50: 728 bp); *Acanthobrahmaea europaea* contained 1803453 reads and 27457 ‘genes’ (N50: 850 bp); *Hepialus californicus* contained 1578453 reads and 24882 ‘genes’(N50: 659 bp); *Spodoptera littoralis* contained 1316901 reads and 15165 ‘genes’ (N50: 648 bp). The results were verified by blasting proteins deduced from contig assemblies against raw reads using the local TBLASTN function of BioEdit^18^. Utilizing previously identified seroins^1,3^ as queries, we found seroin-like cDNAs in the transcriptomes of all examined species. The sequences were deposited in GenBank. Accession numbers are in Table S1.

### The phylogenetic analysis of seroins

The sequences of putative seroin proteins were used to construct tentative phylogenetic trees. The sequences of amino acids were aligned with the MUSCLE program^10^ and analyzed with MEGA7 software^19^. Final alignment used for the subsequent phylogenetic analysis is shown in Fig. S4. Blosum 62 + G + F was chosen as the best model for phylogenetic reconstruction in SMS (Smart Model Selection^20^, according to the lowest BIC score (Bayesian Information Criterion) and AIC score (Akaike Information Criterion). Phylogenetic analysis was performed in PhyML 3.0^21^ using the ML method and also the BI method implemented in MrBayes v.3.2.6^22^. Phylogenetic trees were finalized with the aid of MEGA7 software.

## Supplementary information

Supplementary Information

## Data Availability

All data generated or analyzed during this study are included in this published article (and its Supplementary Information files).
