# Supplementary material for: Modular structure, sequence diversification and appropriate nomenclature of seroins produced in the silk glands of Lepidoptera
Source: Sci Rep. 2019 Mar 7;9:3797. doi: 10.1038/s41598-019-40401-3 (PMC6405961; doi:10.1038/s41598-019-40401-3)
Supplement: Supplementary file 1 — Supplementary Information [file 41598_2019_40401_MOESM1_ESM.pdf]

Supplementary Information

Modular structure, sequence diversification and appropriate nomenclature of seroins produced in the silk glands of Lepidoptera

Lucie Kucerova[1], Michal Zurovec*[1,2]Barbara Kludkiewicz[1], Miluse Hradilova[3], Hynek Strnad[3], and Frantisek Sehnal*[1,2]

Affiliations:
[1]Institute of Entomology, Biology Centre CAS, Branisovska 31, 370 05 Ceske Budejovice, Czech Republic;
[2]Faculty of Science, University of South Bohemia, Branisovska 31, 370 05 Ceske Budejovice, Czech Republic;
[3]Institute of Molecular Genetics CAS, Videnska 1083, 142 20 Prague 4; Czech Republic;

*Correspondence to zurovec@entu.cas.cz and sehnal@bc.cas.cz

Contents:

Table S1. The deduced amino acid sequences of seroins and their accession numbers	3
Supplementary figure legends	13
Figure S1. Alignments of amino acid sequences of the Sn1 seroins	15
Figure S2. Alignments of amino acid sequences of the Sn2 seroins	16
Figure S3. Alignment of amino acid sequences of the Sn3 seroins	17
Figure S4. Alignment of 117 seroin protein sequences used for phylogenetic analysis	14
Figure S5. Phylogenetic trees based on 117 seroin protein sequences	18

Table S1. Seroin sequences in species listed in alphabetic order. The first two or three letters and the signs Sn1, Sn2 and Sn3 in the seroin names denote the insect species and the seroin class. In case of gene duplication within class, gene number follows after a hyphen. The splicing versions are distinguished by capital letters inserted after the gene specification. The additional number at the end of the name indicate minor sequence differences (point mutations or short deletions). For detail explanation see section 3.2. * - data on *B. huttoni* were provided by Dr. Arun Kumar K. P., Centre for DNA Fingerprinting & Diagnostics, Hyderabad, India (Singh et al. 2014).

MG604956, **AeSn1A** (*Acanthobrahmea europaea*)
MAFKIIFISITLAVVVNGIKWPGFDDKFGLNFPQIIPNFPSPPPPVLMPKVTFPSISVEKKGKTPGEIFSAVLIT
AYADNKDGEGHKGGGSWLVNDDGHIESGFFGNNLQPNPDLGQSEETETEII

MG604957, **AeSn1B** (*Acanthobrahmea europaea*)
MAFKIIFISITLAVVVNGIKWPGFDDKFGLNFPQDFKPHKMPKFDIPTMDPSFFKHLSPDEIRSHAPGPNEHYSA
SSVSKHTYSSNKNGKKESGGEMNEIYNDNGAVTEKILQFNQKDED

MG649312, **AeSn2A** (*Acanthobrahmea europaea*)
MRSIIFISAVCLQLFWCNGLPNARGVGAYSYSDSAGNRYGGTYGLNDGQVVDVKGDFPPNFHPQNFQDLDDFFPS
YFANLDNVLQETFHRNVEIQRLAFNAARKAFDLTSNQAGYNPYFDSRFPPYNGFGQYGGFPEFPFHRFGIPNNMN
ENSAYAGAFARPGYSRQIAAINPNNPNMPNVDQENRYSESKSGKNPGSNYMSVSSSSYSTSSDTNGQLKSSRGAE
TIVDDNGRITRYKVQS

MG649313, **AeSn2B** (*Acanthobrahmea europaea*)
MRSIIFISAVCLQLFWCNGLPNARGVGAYSYSDSAGNRYGGTYGLNDGQVVDVKGDFPPNFHPQNFQDLDDFFPS
YFANLDNVLQEFPPYNGFGQYGGFPEFPFHRFGIPNNMNENSAYAGAFARPGYSRQIAAINPNNPNMPNVDQENR
YSESKSGKNPGSNYMSVSSSSYSTSSDTNGQLKSSRGAETIVDDNGRITRYKVQS

MG649314, **AeSn2C** (*Acanthobrahmea europaea*)
MRSIIFISAVCLQLFWCNGLPNARGVGAYSYSDSAGNRFPPYNGFGQYGGFPEFPFHRFGIPNNMNENSAYAGAF
ARPGYSRQIAAINPNNPNMPNVDQENRYSESKSGKNPGSNYMSVSSSSYSTSSDTNGQLKSSRGAETIVDDNGRI
TRYKVQS

MG649315, **AeSn3** (*Acanthobrahmea europaea*)
MYKIVTVLVLAVATAQGRPSLPVMDDEIISAVAAGNWDQIRKLIASGLEQDATWKSLTSGNVKDLKAPEGGHVYG
EAEYTFHSATNFNGQTNEQSG

GBCW01005735 (translated to AA), **AsSn3** (*Agrotis segetum*)
MAKFAPIFLLVGVFIRVQALPLTQEEILEAARKSQFEQLQQLLRDNFAKNADFKNQPVIFDGVRNLKPVGGGHVY
GEAEYSFHSASNIDGKTKEDHAGHKVINNDGKVKEFDFTPKAGLLPALPGFKPPSQFGNDYSASDSSAKYASSTA
DKPGDFVKQPVLPSNIPFPPLSFTDIKDLKPTQGGKVFGEAEFQYHTATNVDGKVSEDRGGHKVINNNGQVQEFD
LTPVPQKSLVGSYSRN

XP_013188735, **AtSn1A** (*Amyelois transitella*)
MASTILLIVSFIAATNADYVWVDEESDAPQGYKRMYVPPLPQPPPLPGFGQLPPLPQPPPLYVPDLNQGFQHFQP
NFVPINIPSPADIKNTKPAPGQVFNGVSIKSVSGVSVDKDGNIVKTGGTTFVVNDNGEVKETKVGKNPPNIEDPF
VLAISSEKPHFQMPKIPDPVTFEPITFKPIVFKPFKPITFKPITFEPITFDPITFKPIDMNYKPADGENYVAVSH
SSHVETSNIDGVVSNSGGSSDMVNVNGNTDKELVEFKNDPKHQTEEAEHRNEE

XP_013188737, **AtSn1B** (*Amyelois transitella*)
MASIIILIISLVAFVNAGYVWVDDEEEAPAPPGYKRMYTPPLMQPPPLPGFGYLPPLPQPPPLYVRDFSQGFQHF
PSPFASVNIPSPADIKNIKPEPGQVFSGVSLKSTRGFDKDKDGNTVKTGGTTIVVNDNGEVKETKVGKNPPNIED
PIVYPSIPEPVKIKRKPHVFKPHVFKPFEPITFKPIDMNYKPADGENFVAVSHSTHSESSNINGVVSNSGGSNDM
VNLNGETDREAVEFVNQDDPQNTSAEKNDN

XP_013188736, **AtSn1C** (*Amyelois transitella*)
MHTANSFHWTSHYGHIHGNSADYVWVDEESDAPQGYKRMYVPPLPQPPPLPGFGQLPPLPQPPPLYVPDLNQGFQ
HFQPNFVPINIPSPADIKNTKPAPGQVFNGVSIKSVSGVSVDKDGNIVKTGGTTFVVNDNGEVKETKVGKNPPNI
EDPFVFPTPHFQMPKIPDPVTFEPITFKPIVFKPFKPITFKPITFEPITFDPITFKPIDMNYKPADGENYVAVSH
SSHVETSNIDGVVSNSGGSSDMVNVNGNTDKELVEFKNDPKHQTEEAEHRNEE

XP_013188734, **AtSn1D** (*Amyelois transitella*)
MHTANSFHWTSHYGHIHGNSADYVWVDEESDAPQGYKRMYVPPLPQPPPLPGFGQLPPLPQPPPLYVPDLNQGFQ
HFQPNFVPINIPSPADIKNTKPAPGQVFNGVSIKSVSGVSVDKDGNIVKTGGTTFVVNDNGEVKETKVGKNPPNI
EDPFVLAISSEKPHFQMPKIPDPVTFEPITFKPIVFKPFKPITFKPITFEPITFDPITFKPIDMNYKPADGENYV
AVSHSSHVETSNIDGVVSNSGGSSDMVNVNGNTDKELVEFKNDPKHQTEEAEHRNEE

XP_013188731**, AtSn2** (*Amyelois transitella*)
MNYYFIVLGVICLQTKWIYGIPVGVSSFSYQDSSGNRYGGTYQIPDGQFGHFFAPNDLPFQIPFEQNNYLPQGNF
FPDYFRNFEYLIQEAFRSNFANQNLALKAARKAFDLTSHQAGYIPYFPFRSGDFEDSQMFNSHNSAFAGAAAGPG
YTHQIAAINPGNPNQPNVNVLERFDDQPSIPGSNFVSMSSKSYAMSSNINGKETSNRGSETVVNNNGKITEYSVH
S

XP_013333213, **AtSn3A** (*Amyelois transitella*)
MSKIVILLLLVTVACQGFPTDPNEDLATLAARGDWDAVHRLINTRFSRKDIWAPSPESTGNVKSLKPVEGGEVYG
EAEYTFHSSSNVNGQKTESSGGHKVINDNGRIQEFDFQPKY

XP_013188666, **AtSn3B** (*Amyelois transitella*)
MRSFSLVFLIAACIGSNKVNVMAQFDFFGITIPKFTEFTVPTLPKINIPPINPNTVKNMKPVNGESLNAVFVSST
SSQKNVDGTVQNTSNVKVINNSNGNVTEYSFKN

MG604949, **AkSn1A** (*Anagasta kuehniella*)
MASAVLLIVV**C**VAVVNAGYVWVEEEDDAPPGYKRFQQEFPKNPDPVKFKPFVFKPFKPITFKPFEPITFKPIDIN
YKPGEGETYVAQSHSTHSESSNVNGVEKNSGGSTAMLNINGDTEKEAVEFRDDPKNAKEEEDDE

MG604947, **AkSn1B** *(Anagasta kuehniella)*
MASAVLLIVVFVAVVNAGYVWVEEEDDAPPGYKRNFPEFASINIPSPGDIKNIKPEPGRVFNGVSVKSVSNYEKD
EDGEPIRTGGTTFIVNENGEVKETKVGKNPPNIEDPIVFPSSFQQEFPKNPDPVKFKPFVFKPFKPITFKPFEPI
TFKPIDINYKPGEGETYVAQSHSTHSESSNVNGVEKNSGGSTAMLNINGDTEKEAVEFRDDPKNAKEEEDDE

MG604948, **AkSn1B2** (*Anagasta kuehniella*)
MASAVLLIVVFVAVVNAGYVWVEEEDDAPPGYKRNFPEFASINIPSPGDIKNIKPEPGRVFNGVSVKSVSNYEKD
EDGEPIRTGGTTFVVNENGEVKETKVGKNPPNIEDPIVFPSSNPDPVKFKPFVFKPFKPITFKPFEPITFKPIDI
NYKPGEGETYVAQSHSTHSESSNVNGVEKNSGGSTAMLNINGDTEKEAVEFRDDPKNAKEEEDDE

MG604946, **AkSn1C** (*Anagasta kuehniella*)
MASAVLLIVVFVAVVNAGYVWVEEEDDAPPGYKRMYVPPLQQPPPLPPGFGVLPPLSQPPPLYVRDFSKPLPEFQ
HFQNFPEFASINIPSPGDIKNIKLEPGRVFNGVSVKSVSNYEKDEDGEPIRTGGTTFVVNENGEVKETKVGKNPP
NIEDPIVFPSSFQQEFPKNPDPVKFKPFVFKPFKPITFKPFEPITFKPIDINYKPGEGETYVAQSHSTHSESSNV
NGVEKNSGGSTAMLNINGDTEKEAVEFRDDPKNAKEEEDDE

MG604950, **AkSn2** (*Anagasta kuehniella*), partial sequence
HISGTPVGVSSFAYQDSSGNSITQTSDFSRSAESRFGLPPLRPLKFLKPLSRSSDFGNFGNMFNSPNSAFAGGFA
SPGFTHQIAAISPPNPSNPNVNVFERFNEGSAIPGANFVSMKVQPTTSSSNVNGKEISNRGSENCRQINNGKVTG
YSVHS

DQ666525, **AmSn1** (*Antheraea mylitta*)
MALTKIFLALSLVALSNAVLMWPNDDDRFPPLPRNNIRRYPSRGFPLFPDFQSVLSFPFNFVGGGGFPGGGAFAG
GGAFAGGGGFAGDGGFTSGGVFSSIPFPSPSDITNTKPAPGQTYTGIFAHSGGGEHYIMANLNGHVVKYSDDGET
ASETVINK

MG604951, **AySn1A** (*Antheraea yamamai*)
MALTKIFLTLSLVALSNAVLMWPNDDGRFPPLPQNNYRRYPSRGLPLFPDFQSVLSFPFNFVGGGGFPGGGAFAG
GGAFAGGGGFAGDGGFTSGGVFSSIPIPSPSDITNTKPAPGQTYTGIFAHSGGGEHYIMANLNGHVVKYSEDGET
ASESVINK

MG604952, **AySn1B** (*Antheraea yamamai*)
MALTKIFLTLSLVALSNAVLMWPNDDGRFPPLPQMNMFFPDPIKFTMPRIVPFHIPPPPIMDRDAIKTHVAGPNE
HFTGFSTSSHSFTSNDNGKITSGGGISTLTNDGKNVEENVLEYGDKGNNDNNNNNNNNNN

MG604953, **AySn1C** (*Antheraea yamamai*)

MALTKIFLTLSLVALSNAVLMWPNDDGRFPPLPQNNYRRYPSRGLPLFPDFQSVLSFPFNFVGGGGFPGGGAFAG
GGAFAGGGGFAGDGGFTSGGVFSSIPIPSPSDITNTKPAPGQTYTGIFAHSGGGEHYIMANLNGHVVKYCMY

MH127913, **AySn2A** (*Antheraea yamamai*)
MGTHCFSVQYVAPTSPSGVPDQSGVGAFSYQDSAGNRYGGTYGLRDGQVVDTKGDFPPNFHFNTFQDLDSFFPEY
FRNLGNLLQEAFNANFENQRLAFNAAQKAFDLTSNQAGYNPNFNYPGAGNFPNFGDFPGVPLAFPAMPNFGNEFG
AFAAAVANPGYRQQIAAINPSNPGVPNVNEVRYSNSETPGNNGGYMAVSSTSYSTSSQNADGQITNSRAAETVIN
DNGKITKYSVKN

MH127914, **AySn2B** (*Antheraea yamamai*)
MGTHCFSVQYVAPTSPSGVPDQSGVGAFSYQDSAGNRAFNANFENQRLAFNAAQKAFDLTSNQAGYNPNFNYPGA
GNFPNFGDFPGVPLAFPAMPNFGNEFGAFAAAVANPGYRQQIAAINPSNPGVPNVNEVRYSNSETPGNNGGYMAV
SSTSYSTSSQNADGQITNSRAAETVINDNGKITKYSVKN

MG604955, **AySn3** (*Antheraea yamamai*)
MSKLIIVVVMAVAFYVTEANPAPNAFDPINNFAPFNPRHFPIAHSFESVRALRPEEGGQVSGMAISTSTRDDGAG
GSVSQGRVLVNKNGKLYETSFRKK

BhSn1-1* (*Bombyx huttoni*)
MAFTKFLFVVTLITIASGASFVWEDDDDMFPGVCIVFLQFKMPEMKPLEFDDIKSHVAGDNEHYTGESISSYSSS
STVNGKKESSGGVSVLTNDGKAVKEKVMEYKDGD

BhSn1-2* (*Bombyx huttoni*)
MAFTKFLFMLSLITIASAGFVWQDDNFPGFPSDMWPSIQIPTIPPFDPKIPNFAFSFPSPDNIKKTKPQPGQTYS
GVYVSSNGGKGTMVANINGEVIEKKFGEDSKKSKSKS

NP_001037045, **BmSn1-1** (*Bombyx mori*)
MAFTKFLFVITLITIASAGFVWEDDDDLFPGFSDAFKMREIPEIKSLEFDDIKTHVAGDNEQYTGESKSSYSSSS
TVNGKTVSSGGVSELTNDGKAVEEKVMEYKDGD

NP_001037046, **BmSn1-2** (*Bombyx mori*)
MAFTKFLFMLSLITIASAGFVWQDDNFPGFPSDMWPSIQIPTIPPFDPKIPNFAFSFPSPDNIKKTKPQPGQTYS
GVYVSSNGGKGTMVANINGEVIEKKFGEDSKKSKSKS

XP_004932380, **BmSn2A** (*Bombyx mori*)
MRNILMVCAICLVLAVGNALPNNRGVGAFAYQDSSGNRYGGTYGLKDGAVVDKQGDFPPNFQPESFQDLDYFFPE
YLRNFENLLREPFFRFTPIRTKSFFPYTPLRFEPFRPFPSYPQRAFSSNIEAQRLAFEAAQKAFDLTSNQAGYIP
GFDRFPIFNFPSPGFTDEQFPAMQNYPNQFGAFAGAAAGPGFSHQVASINPPNPNMPNVNKYTSYSGAQPNDGNQ
FVSVSSSSYSSSVNDNGEVKNHRAAETVVNNNGKVTKYKVEN

XP_004932382, **BmSn2B** (*Bombyx mori*)
MRNILMVCAICLVLAVGNALPNNRGVGAFAYQDSSGNRYGGTYGLKDGAVVDKQGDFPPNFQPESFQDLDYFFPE
YLRNFENLLREPFFRFTPIRTKSFFPYTPLRFEPFRPFPSYPQRFPIFNFPSPGFTDEQFPAMQNYPNQFGAFAG
AAAGPGFSHQVASINPPNPNMPNVNKYTSYSGAQPNDGNQFVSVSSSSYSSSVNDNGEVKNHRAAETVVNNNGKV
TKYKVEN

XP_004932383, **BmSn2C** (*Bombyx mori*)
MRNILMVCAICLVLAVGNALPNNRGVGAFAYQDSSGNRPFFRFTPIRTKSFFPYTPLRFEPFRPFPSYPQRAFSS
NIEAQRLAFEAAQKAFDLTSNQAGYIPGFDRFPIFNFPSPGFTDEQFPAMQNYPNQFGAFAGAAAGPGFSHQVAS
INPPNPNMPNVNKYTSYSGAQPNDGNQFVSVSSSSYSSSVNDNGEVKNHRAAETVVNNNGKVTKYKVEN

XP_004932379, **BmSn3** (*Bombyx mori*)
MKLVLVTVLIVVSVQAVPSFPIGDELFDAASSGDWEKVHELINSKLNENDSWKPVSAGSVKSLKPIPGGHVYGES
EYTFHSSSDINGQKTDKSGGHKIINDDGKVYEFDFNPKVKGY

GADL01001805 (translated to AA), **CsSn1** (*Carposina sasakii*)
MTYNFFTCAAFVVVVVHAGAAWQQANAWSGFQFPQMPNFILPTPPTPPTPPTLPTFPTLPFAPFRPFAPFLPFAP
VAPIAPVILPPLRPADIINMVPGPGQSFNGISVSSVSGVTKDKDGNIVKTGGTTILTNEDGVTQEFKVGENPPDI
NKPIVVPPLPTPPKLPTLPKLPSLPTLPTLPNFEPIEVKIPEFNPIEIDVAPGGENHFSTFSSSSFSHSSDINGE
KTAGGGHKIYVNVDGEVQQEGSLYGVN

FC974337 (translated to AA), **CfSn1A** (*Choristoneura fumiferana*), partial sequence
PGQGQVFHGVAAQSSSGMVQDKDGKWVRAGGNSVLANDNGKVQELKQGEAPPDLNVPIRAPTMPKMPELKFTHLP
KIKPMEPIKFEPITFKSFKPITFKPIVFEPIPPIDTEKLKTYQPKEGEQFFGSAVSSFSSSETVNGKTAAEGGGS
IIVNDNGKADEQHVVFHEGDKDFAEE

FC967135 (translated to AA), **CfSn1B** (*Choristoneura fumiferana*), partial sequence
AVGAHAKFVWQNDNEWPAFQQIRMPKMPEMKPFKFEPITFKPIVFEPIPPIDTEKLKTYQPKEGEQFFGSSVTSF
SSSKTVNGKTVSEGGGSVIVNDNGKADEQHVVFHEGDKDFAEE

MG649316, **CpSn1A** (*Cydia pomonella*)
MASVRILIVAAVLVGCANAKFVWEPDTNDDFPAFKPFKMQMPVPPTPPQLPFPFPSFQNFAPFQPIRPIQPFAPN
AFNFPMPHIISADEIKNAKPGPNGVYNGVMVSSSSNSYVDKDGKVVKKGGTSVLTNQDGKVQEWKQGNAPPDLNK
PIQVGPLW

MG649317, **CpSn1B** (*Cydia pomonella*)
MASVRILIVAAVLVGCANAKFVWEPDTNDDFPAFKPFKMPKFEPITFDPIEPIVFEPIEFEPIKFDPIEPIKIDD
IKNHKPGKGEKYSGYSVSASKSTKTVNGKVVEDKAASSVLTNDNGKVDQQHAGYNKGEKEPRRNK

MG649318, **CpSn1B2** (*Cydia pomonella*)
MASVRILIVAAVLVGCANAKFVWEPDTNDDFPAFKPFKMPKFEPITFDPIEPIEFEPIKFDPIEPIKIDDIKNHK
PVKGEKYSGYSVSASKSTKTVNGKVVEDKAASSVLTNDNGKVDQQHAGYNKGEKEPRRNK

MG649319, **CpSn1C** (*Cydia pomonella*)
MASVRILIVAAVLVGCANAKFVWEPDTNDDFPAFKPFKMQMPVPPTPPQLPFPPFPSFQNFAPFQPIRPIQPYAP
NAFNFPMPHIISADEIKNAKPGPNGVYNGVMVSSSSNSYVDKDGKVVKKGGTSVLTNQDGKVQEWKQGNAPPDLN
KPIQMPKFEPITFDPIEPIVFEPIEFDPIEPIKIDDIKNHKPGKGEKYSGYSVSASKSTKTVNGKVVEDKAASSV
LTNDNGKVDQQHAGYNKGEKEPRRNK

MG649320, **CpSn3** (*Cydia pomonella*)
MPKFTFVLVIVALAISVQGFPTFEDDVAAAAASGDWAKVHEILRKNFASSFGQNTMTDVRNLKPQNGGHVFAEAK
STFEHSSNINGKSSHERGGHEVVNNDGKVSEWDLN

OWR44300, **DpSn2** (*Danaus plexipus*)
MKVLLISAILLQAWCNAYPKPKGVASYVYTDTDGNRYGGTYDLGNNIAPFSDPIISSFSDPYVPEFFNNFGNILP
QIDLKNQRPALLNDPRAFNPTYQRRGYNPSFSRFPFGGFPFGPSAFNVNRFGENAAFASSAIAPGYRHQSAIISP
ANADIPNVSLTDRYADPSDDGKFYSVSSSSFASSDNDNGKLSGFRQAETVVNDNGKITKYRVHS

OWR47400**, DpSn3** (*Danaus plexipus*)
MYKLIIVFVVSAAVLAEGRPSLAKELVESAKSGNWDLFHELVRQQHALADWAFDFTSDVENLKPVDGANVSGHSI
IVTKQWSDDGTNHSEHGEGREVINDNGQVTVRRFQI

MG604938, **GmSn1A** (*Galleria mellonella*)
MATKILIFLSFVALSSAGFVWVDDDNNSFPKLRQLYVPPLPQPPPLPNIPGLPQPPPLPQPPPLFGFDFSPILPI
PPIPPIPPILPTPPFINIPAPEDIKNIKPKPGQFFNGISVKSRSGYALDKDGNRVKTGGTAVLINDNGEVNETIV
GDNPPKFEESRKESSSN

MG604939, **GmSn1B** (*Galleria mellonella*)
MATKILIFLSFVALSSAGFVWVDDDNNRFPKLRQPIFPKIPKFEPFTFKPFVFKPFVFEPITFKPIDVNYQPKEG
ENFVAVSTSSHHESSNVNGVEKSSGGSDIVTNVGGKINEEGVEFKKGDRDDENNNEERNTNENSGENNE

MG604940, **GmSn1B2** (*Galleria mellonella*)
MATKILIFLSFVALSSAGFVWVDDDNNRFPKLRQIPKFEPFTFKPFVFKPFVFEPITFKPIDVNYQPKEGENFVA
VSTSSHHESSNVNGVEKSSGGSDIVTNVGGKINEEGVEFKKGDRDDENNNEERNTNENSGENNE

MG604941, **GmSn1C** (*Galleria mellonella*)
MATKILIFLSFVALSSAGFVWVDDDNNRFPKLRQLYVPPLPQPPPLPNIPGLPQPPPLPQPPPLFGFDFSPILPI
PPIPPIPPILPTPPFINIPAPEDIKNIKPKPGQFFNGISVKSRSGYALDKDGNRVKTGGTAVLINDNGEVNETIV
GDNPPKFEESRKESSSIFPKIPKFEPFTFKPFVFKPFVFEPITFKPIDVNYQPKEGENFVAVSTSSHHESSNVNG
VEKSSGGSDIVTNVGGKINEEGVEFKKGDRDDENNNEERNTNENSGENNE

MG604944, **GmSn2A** (*Galleria mellonella*)
MGSVLSGALLITRPGAPEGVGAYAYQDSAGNRSPFNNFPGFGVIPSFPTFGMSNFPNSAFAGGAAGPGFTHQIAS
INPSNPDMPNVDVMNRFADVSQTPGGNYVAMSSRAYSVSSNVNGKEYKDRGAETTVNNNGKITTYRVKS

MG604943, **GmSn2B** (*Galleria mellonella*)
MGSVLSGALLITRPGAPEGVGAYAYQDSAGNRAFHSNLENQRIALNAASKAFDLTSNQAGYIPSFPHRSPFNNFP
GFGVIPSFPTFGMSNFPNSAFAGGAAGPGFTHQIASINPSNPDMPNVDVMNRFADVSQTPGGNYVAMSSRAYSVS
SNVNGKEYKDRGAETTVNNNGKITTYRVKS

MG604942, **GmSn2C** (*Galleria mellonella*)
MGSVLSGALLITRPGAPEGVGAYAYQDSAGNRYGGTYGLNDGQFFGQKGQHFPIPFAQNNFQNNFLDLDDFFPEY
FRNFENLLQEAFHSNLENQRIALNAASKAFDLTSNQAGYIPSFPHRSPFNNFPGFGVIPSFPTFGMSNFPNSAFA
GGAAGPGFTHQIASINPSNPDMPNVDVMNRFADVSQTPGGNYVAMSSRAYSVSSNVNGKEYKDRGAETTVNNNGK
ITTYRVKS

MG604945, **GmSn3** (*Galleria mellonella*)
MSRLTVVFVLATFLCVNIANANEFPFFGGNFPFPSFDFPKINIPKIKPINIDDIKNLKPSDGGVVNGAAVSSSST
VENVNGVPVRKQQTRIITNDNGQVKEVTYDN

GADK01000222 (translated to AA), **GmoSn1** (*Grapholita molesta*)
MASATFLIVVAVLVGCANGKFVWQSDDNDDFPAFKMQMPVPPTPPKLPFAPFPPFQPFAPFQPFAPFQPVQPFFP
FPPNAFKVPMPRILTADEIRNAKPGQNGVYNGIMVSSSSSSYVDKDGKVVRKGGTSVLSNQDGKVKEWKEGNAPP
DLNKPVQVRQPW

HMEL015978, **HmSn1**(*Heliconius melpomene*)
MHDFSVYFQLLSLSEVELLLLYQCQIMAYTILLFTAFIAASVNEDMNLRMPPIPPIVIPTPEDIVKNKGPNFNGI
SVSSSSSSTVDKDGKVIQTGGTTVVTNNDGVVKEFVFGDNPPNVISASSSFTQATNINGVKTSVGGGSLVSNVNG
DVQENINYFGSNTV

AHX25884, **HaSn1**(*Helicoverpa armigera*)
MAVTTVLIVASLVAIASAGFPGFPWPENDDVFPPLPVPFFEFDAPQPKPLNHNNRFKKFEFNFEPLNPERLKKIV
PGENEYFKGKSVRSHSYSSNVNGVVNQEASVIRVENDNGKVNEETISFRKGPER

XP_021200362, **HaSn2A** (*Helicoverpa armigera*)
MMKIIFLVFIPLAWSYGPNTQGGGAFAYVDSTGNRYGGTYGLEDGKVVRTSGDPIPEHFADTVDPYHGADFGPLF
FGNFDHLLQEVFQKNFENQRLAFDAARQAFDLSSNQLGGYYPNPNVDYRYPPFGGFGMPMSGFGNSAFASASAGP
GYQHHVAAISPSNPRMPNVDRVSHFADTSLPDGRKYYSVSSKSYSSSSNINGREISNRGAETLVNDNGKVTHYKV
QN

XP_021200363, **HaSn2B** (*Helicoverpa armigera*)
MMKIIFLVFIPLAWSYGPNTQGGGAFAYVDSTGNRYGGTYGLEDGKVVRTSGDPIPEHFADTVDPYHGADFGPLF
FGNFDHLLQEYPPFGGFGMPMSGFGNSAFASASAGPGYQHHVAAISPSNPRMPNVDRVSHFADTSLPDGRKYYSV
SSKSYSSSSNINGREISNRGAETLVNDNGKVTHYKVQN

XP_021200479, **HaSn3** (*Helicoverpa armigera*)
MAKFASTLILVVAIISVQGGPVTYDEILAAARQQQFDKVQEFPQSETDSEDEMQLRSNFGKNTEVWKPSFNSVRD
LKPADGAHVYGEAEYSFHSASNIDGKTSEEHAGHKIINDDGKIEEFDFTPSSTQFPLLSPIITSLGGHSPISNDY
GADYSFHHVPVGVGEMSEYISGKKVDSKNKNIALSALKLPITPISFTGVRDLKPEKGGQVYGEAQYAYRSATSVN
GKTTEDKGGHKIINNNGKVKEFDFTPSADQTNVSFF

GBTA01037100 (translated to AA), **HasSn3** (*Helicoverpa assulta*)
MSKFASTLILVVAIISVQGGPVTYDEILAAARQQQWDKVQELLKSNFGKNTEVWKPSFNSVRDLKPADGAHVYGE
AEYSFHSASNIDGKTSEEHAGHKVINDDGKVEEFDFTPSSTQFPLLSPIITSLGGHSPISSDYGGADYSFHHVPV
GVGDTSEYISGKKVDSKNQNTALSALKLPLTPLSFTGIRDLKPEKGGQVYGEAQYAYRSATNVNGKTTEDKGGHK
IINNNGKVKEFDFTPSADQTNVSFF

MG649321, **HcSn1A** (*Hepialus californicus*)
MGGFSVIVFAACLLVSNVGVSSGFVFLPGFGFPHPVNAFQQARDIQSFVHNQIRSMTPFAGGVPVPVGPGVHVFN
GPNGAGVVSAAGAGGGVVSTGTGGGTVVSTSTGGGTGGFVSTLGGGGGHYGGGSTFVSSGGPKGGFGVIQTSSNI
DGKQSGQVLINDNGKVTAYELPPGGGTVFTVQSNKGGPGVASSSYSSSYSNGDGEKSSEGGVIDDN

MG649322, **HcSn1A2** (*Hepialus californicus*)
MGGFSVIVFAACLLVSNVGVSSGFPHPVNAFQQARDIQSFVHNQIRSMTPFAGGVPVPVGPGVHVFNGPNGAGVV
SAAGAGGGVVSTGTGGGTVVSTSTGGGTGGFVSTLGGGGGHYGGGSTFVSSGGPKGGFGVIQTSSNIDGKQSGQV
LINDNGKVTAYELPPGGGTVFTVQSNKGGPGVASSSYSSSYSNGDGEKSSEGGVIDDN

MG649323, **HcSn2** (*Hepialus californicus*)
MASASAFASSDSIRNRTPGFASAQASASGTGGIGRSNRAFMNNINNQRAALEASQRAFALAAQGSGGYQGPASFG
YGYNPGYNPGYNPGYNPGYNAGFPQGGFQQAGNQGGAGNPQAAFASASIDPTGTRQIASISPSNSNSPNVDVHGPAPSR
DGGGDGKFYGVSSSSFSSSSNVNGVPTSQRTAHTVINDNGKITEHKVAS

MG652368, **MbSn1A** (*Mamestra brassicae*)
MAFTTLLVTVSMVAIANAG**W**PHNDNPFPGFAGPMPFVFAYPKIPPHNPYQFGYSSFRHSIPFVDPKKIESYVPGP
GETYKGSSIRSSAFSTNYNGKVNQGGSLHMIDNDNGRVKQQHHTFGTPP

MG652369, **MbSn1A2** (*Mamestra brassicae*)
MAFTTLLVTVSMVAIANGLNFGHFFLAGWPHNDNPFPGFAGPMPFVFAYPKIPPHNPYQFGYSSFRHSIPFVDPK
KIESYVPGPGETYKGSSIRSSAFSTNYNGKVNQGGSLHMIDNDNGRVKQQHHTFGTPP

MG652370, **MbSn1B** (*Mamestra brassicae*)
MAFTTLLVTVSMVAIANAGWPHNDNPFPGFAGPMMPSLRFGPLASQSSLPGMPNFGQNFPFFQLPAFPTMPAMTF
PSLPSASDIANAKPGKGGTFTGVVITSKTEAKRKDDGTIVKESGSTILMNNDGVVTVKKCMYLLAMFFWLTNSF

MG652371, **MbSn2A** (*Mamestra brassicae*)
MMKILFVVMLSLAWSYGVPNSEGGGAFAFVDSSGNRYGGTYGLKDGKVVSRTGDPFPQHFAENIPYPNFANNIPY
QNFGDNIPYQNFADTVYPLEEDFGQAYFSNLENLLQEVFNKNLESQKLAFNAARKAFDLTSNQAGGYAGGYSGGY
SGGYYPNPNIASRYPAFPSFPTFTMPRPMNFGNSAYASAAAGPGYQHHVASINPSNPANPNVDRTINHFSDTSAP
RSGFYSVSSNSYSSSSNVNGKELNNRGAETVVNDNGKVTHYKVQS

MG652372, **MbSn2B** (*Mamestra brassicae*)
MMKILFVVMLSLAWSYGVPNSEGGGAFAFVDSSGNRYGGTYGLKDGKVVSRTGDPFPQHFAENIPYPNFANNIPY
QNFGDNIPYQNFADTVYPLEEDFGQAYFSNLENLLQEYPAFPSFPTFTMPRPMNFGNSAYASAAAGPGYQHHVAS
INPSNPANPNVDRTINHFSDTSAPRSGFYSVSSNSYSSSSNVNGKELNNRGAETVVNDNGKVTHYKVQS

KOB72512, **ObSn3** (*Operophtera brumata*), partial sequence
APQLPFEDIIAAANLGNWDKVQSLLSSKFGTAGWQPVPLGNVRSLKPVKGGHVYGESEYTFQSSSDQNGQKTEQS
AGHKIINNDGDVKEFDFKPKFEQNPFFKLS

GAQJ01002982 (translated to AA), **OfSn1A** (*Ostrinia furnacalis*)
MPIMPRLPTLPIMPTPPIPPKAPRFPFGGFQMPFIPLPKIPTAAEMASVKPGPNQVYNGVAVKSSSSFTRDKDGK
LVRTGGTHILVNDNGEVQEEKFGTRPPNLNDPIVFPSMPEFKFSLPPLPPMPPMPPMPPMPAMPPMPAMKPIKPI
IINPLDIEQHQPGENEHFVGVSTSSYSHSSNINGVEKSGGGAHYVVNVDGEVEKEVLHFGQGDKE

GAQJ01002988 (translated to AA), **OfSn1B** (*Ostrinia furnacalis*)
MPEFKFSLPPLPPMPPMPPMPPMPAMPPMPAMKPIKPIIINPLDIELHQPGENEHFVGVSTSSYSHSSNINGVEK
SGGGAHYVVNVDGEVEKEVLHFGQGDKE

MG674516, **OnSn1A** (*Ostrinia nubilalis*)
MARTILIIVSLVAAVHAGFVWTNDNENNFSPFQHIRMPIMPRLPTLPIMPTPPIPPKAPRFPFGGFQMPFIPLPK
IPTAAEMASVKPGPNQVYNGVAVKSSSSFTRDKDGKLVRTGGTHILVNDNGEVQEEKFGTRPPNLNDPIVFPSVR
FY

MG674517, **OnSn1A2** (*Ostrinia nubilalis*)
MARTILIIVSLVAAVHAGFVWTNDNENNFSPFQHIRAPRFPFGGFQMPFIPLPKIPTAAEMASVKPGPNQVYNGV
AVKSSSSFTRDKDGKLVRTGGTHILVNDNGEVQEEKFGTRPPNLNDPIVFPSVRFY

MG674518, **OnSn1B** (*Ostrinia nubilalis*)
MARTILIIVSLVAAVHAGFVWTNDNENNFSPFQHIRMPEFKFSLPPLPPMPPMPPMPPMPAMPPMPAMKPIKPII
INPLDIEQHQPGENEHFVGVSTSSYSHSSNINGVEKSGGGAHYVVNVDGEVEKEVLHFGQGDKE

MG674519, **OnSn1C** (*Ostrinia nubilalis*)

MARTILIIVSLVAAVHAGFVWTNDNENNFSPFQHIRMPIMPRLPTLPIMPTPPIPPKAPRFPFGGFQMPFIPLPK
IPTAAEMASVKPGPNQVYNGVAVKSSSSFTRDKDGKLVRTGGTHILVNDNGEVQEEKFGTRPPNLNDPIVFPSMP
EFKFSLPPLPPMPPMPPMPPMPAMPPIKPIIINPLDIELHQPGENEHFVGVSTSSYSHSSNINGVEKSGGGAHYV
VNVDGEVEKEVLHFGQGDKE

MG674520, **OnSn2A** (*Ostrinia nubilalis*), partial sequence
SLRTGDPAPAGVGAYAYQDSAGNRYGGTYGLDGAKEYSSKGKPIGPGNRGTADPWGNFADAQAFSGGFGGGWPNF
APYQQNFQQYDDFFPEYFNNLQTLLNEAFSSNFENQKLAYNAARKAFDLTSNQAGYIPNFTSRLNNFNFGGFPPF
GMPMPMGPNSAFAGASAGPGFTHQVAALNPENPSEPNVNVMSRFGEDVPRGRGKYVSVSSSSFSSSSNVNGKEKT
HKAAETYVNNNGKVTHYRVES

MG674521, **OnSn2B** (*Ostrinia nubilalis*) , partial sequence
SLRTGDPAPAGVGAYAYQDSAGNSRLNNFNFGGFPPFGMPMPMGPNSAFAGASAGPGFTHQVAALNPENPSEPNV
NVMSRFGEDVPRAGRGKYVSVSSSSFSSSSNVNGKEKTHKAAETYVNNNGKVTHYRVES

MG674522, **OnSn3** (*Ostrinia nubilalis*)
MSKFALLAVLAVLAVAQGYPQKEVEISDAASNGDWNLVHKLIQQRFENQRNLWEPSLTIPTGNVRSLKPLEGGHV
YGESEYTFRSESNVNGKKTESKGGHRVVNKDGVVTEYDIEPKF

MH127915, **OsSn2** (*Oligotricha striata, Trichoptera*), partial sequence
LYIHARGGNNDGAASASASFGPGGGHQSANIYPENPSNPNINNRFGSPGTKPGGEYGVSTSSFSSSSNVNGKKSS
HKQASTSINDNGKVTTFVVRDSLFWPV

XP_014366087**, PmSn1A** (*Papilio machaon*)
MAYFFLIATIVLVVGVNADPSNDNQNPFQGFPFDQFQKPFQNIHVNSFQPFFPFPLHPIIPPFPTIKIPSPDDII
GKKPSPGESFNGVLVQSSSGFTTDENGNVIKNGGTTVLTNDNGEVKEFKVGKTPPVIKSNIVPPFQNPKITITKG
KMPVMPPMPLFFPQIHITSFNNDNLKRIKPGPNQHFVGSSSSSFTVSSNVNGRKNYAGATKTIVNNNGHVDEQAL
IIENSV

XP_014366086**, PmSn1B** (*Papilio machaon*)
MAYFFLIATIVLVVGVNADPSNDNQNPFQGFPFDQFQKPFQNIHVNSFQPFFPFPLHPIIPPFPTIKIPSPDDII
GKKPSPGESFNGVLVQSSSGFTTDENGNVIKNGGTTVLTNDNGEVKEFKVGKTPPVIKSNIVPPFQNPKITITKG
KTPPINLSKLPKIDLPKLPDINIPGIINIKGVQLPSIDFSQLPDIPESEMPVMPPMPLFFPQIHITSFNNDNLKR
IKPGPNQHFVGSSSSSFTVSSNVNGRKNYAGATKTIVNNNGHVDEQALIIENSV

KPJ09383, **PmSn2** (*Papilio machaon*)
MAFLAIENQFNDHDTKRSGSPNPDGVGVFAYQDSAGRRYECVYKPNVNEADDSSESSNVNKINEVLPYDTNWPCR
RFLPPLPPLPPLKPLTPFKPLRPLPPLKPLPRLLSPFPTLSPFPQLPPLPHFKKLRDVIFGNIENQQRAAFEAAQ
NTFGAFNNFPFIPNFDFRYPPYGHFNNFGPSYRAPFDMPGSNSAFAAGAVGPGFRHQVAAINPGNPQMPNVDTTM
NREPENRQGFYSVSSSSYASSLNNNGVPQNQRGAETVVNDNGRITKYVLGIPGCARQEDVVCRSRRTAYCGRHWE
RPTSTTEQTKDNDDDTEVLTSNVMILKI

XP_014366094**, PmSn3** (*Papilio machaon*)
MMKLCPVIFVIGFLFINVEGAPFDANDISAAAANGDWDTFHRLIRNKFLLAHNRIEDKFRSMRRRLSQGFDFKPN
FESIRSLQPDGPDSNVYGEAQYSFHSSSNVNGQKSEQRGGRRIINKNGVVEEYELP

XP_013149659, **PpSn1** (*Papilio polytes*)
MAYFTLIATIVLVVGVNADSSDNDNPFPGFPFNQFQRPFHNVHVNSFQPFFPFPLHPVIPPFPTIKIPSPDDIIG
KNPNPGETYSGIVVQSSSGYTTDENGNVVKTGGTTVVTNDNGEVKEYKVGKNPPVIKNNIVPPVPVVPPMPLFFP
EIHISSFNNDNLKNIKPGPNQHFVGSSSSSFTVSSNVNGKKNYAGASKTIVNNNGKVDEQALVIENSV

XP_013149635, **PpSn2A** (*Papilio polytes*)
MKVVYLVSAIVLQCSWSYGSPGPDGVGVFAYQDSTGRRYECVYKPNINEADDSSESSNVNKINEVLPFDTNWQCR
RFLPPLPPLPPLKPLTPFKPLRPLPPMKPLPRLLSPFPTLSPFPQLPPLPHFKTLRDTIFGNIENQQRAAFEAAQ
NSFGAFNNFPFIPNFDFRYPPYGHFNYFGPSYRAPFDMSGSNSAFAAGAVGPGFRHQVAAINPGNPQMPNVDTTM
NRAPENRQGFYSVSSSSYASSMNNNGVPQNQRGAETVVNDNGRITKYVVHN

XP_013149637, **PpSn2B** (*Papilio polytes*)
MKVVYLVSAIVLQCSWSYGSPGPDGVGVFAYQDSTGRRYECVYKPNINEADDSSESSNVNKINEVLPFDTNWQCR
RFLPPLPPLPPLKPLTPFKPLRPLPPMKPLPRLLSPFPTLSPFPQLPPLPHFKTLRYPPYGHFNYFGPSYRAPFD

MSGSNSAFAAGAVGPGFRHQVAAINPGNPQMPNVDTTMNRAPENRQGFYSVSSSSYASSMNNNGVPQNQRGAETV
VNDNGRITKYVVHN

XP_013149638, **PpSn2C** (*Papilio polytes*)
MKVVYLVSAIVLQCSWSYGSPGPDGVGVFAYQDSTGRRYECVYKPNINEADDSSESSNVNKINEVLPFDTNWQDT
IFGNIENQQRAAFEAAQNSFGAFNNFPFIPNFDFRYPPYGHFNYFGPSYRAPFDMSGSNSAFAAGAVGPGFRHQV
AAINPGNPQMPNVDTTMNRAPENRQGFYSVSSSSYASSMNNNGVPQNQRGAETVVNDNGRITKYVVHN

XP_013149639, **PpSn2D** (*Papilio polytes*)
MKVVYLVSAIVLQCSWSYGSPGPDGVGVFAYQDSTGRRYECVYKPNINEADDSSESSNVNKINEVLPFDTNWQYP
PYGHFNYFGPSYRAPFDMSGSNSAFAAGAVGPGFRHQVAAINPGNPQMPNVDTTMNRAPENRQGFYSVSSSSYAS
SMNNNGVPQNQRGAETVVNDNGRITKYVVHN

NP_001298377**, PpSn3** (*Papilio polytes*)
MKLCPVIFIIGFLFFSVEGAPFNQNEINAAAANGDWETFHRLLSQGFDFKPNFDSIRSLQPDGPDSKVYGEAQYS
FHSSSNVNGQQSEQRGGRRIINKNGVVEEYELP

XP_013177374, **PxSn1A** (*Papilio xuthus*)
MAHFFLIATIALLVGVNADPSNDNQNPFQGFPFNQFQRPFQNFHVNGFQPFFPFPLHPIIPPFPTIKIPSPDDII
GKNPSPGETFSGVVVQSSSGYTTDENGNVIKTGGTTVLTNDNGDVKEFKVGKNPPVIKSNIVPPVPVVPPTPVFF
PQIHITSFNSDNLKNIKPGPNQHFVGSSSSSFTVARNVNGKKNYAGATKTIVNNNGQVDEQALIIENSV

KPI97590, **PxSn1B** (*Papilio xuthus*)
MAHFFLIATIALLVGVNADPSNDNQNPFQGFPFNQFQRPFQNFHVNGFQPFFPFPLHPIIPPFPTIKIPSPDDII
GKNPSPGETFSGVVVQSSSGYTTDENGNVIKTGGTTVLTNDNGDVKEFKVGKNPPVIKSNIVPPVPVVPPTPVFF
PQIHITSFNSDNLKNIKPGPNQHFVGSSSSSFTVARNVNGKKNYAGATKTIVNNNGQVDEQALIIENSVPVVPPT
PVFFPQIHITSFNSDNLKNIKPGPNQHFVGSSSSSFTVA**S**NVNGKKNYAGATKTIVNNNGQVDEQALIIENSDND
NKEQEASDSSYMKQNMEAPASYIPLYYGYVPFYELIGY

XP_013177102, **PxSn2** (*Papilio xuthus*)
MKFLYLVSAVVLHCSWSYGSPSPDGVGVFAYQDSTGRRYECVYEADDSSESSNVNKINEVLPFDTNWPCRRFLPP
LPPLPPLKPLTPFKPLRPLPPMKPLPRLLSPFPTLSPFPQLPPLPHFKKFRDVIYDNIENQQRAAFEAAQNTYGA
FNNFPFIPNFDFRYPPYGHFNYFGPSYRAPFDMSGSNSAFAAAAVGPGFRHQVAAINPGNPQMPNVDTTINRQPQ
NRQGQGFYSVSSSSYASSLNNNGVPQNQRGAETVVNDNGRITKYAVHN

XP_013177509, **PxSn3** (*Papilio xuthus*)
MMKLCPVLFVIGFIFINVEGAPFDQNDISAAAANGDWNTFHRLIQNKFRLARNRIEDKFKLMRRRLSQGFDFKPN
FDSIRSLQPDPDSNVYGEAQYSFHSSSNVNGQKSEQRGGRRIVNKNGVVEEYELP

PIENAPT00000019993, **PnSn1A** (*Pieris napi*)
MALRCVVAFLAVTCTHALNQYQNEYGNFQNNHGFRQPQFPSITMPPIKPIVFPTFPTFSPEDIINQRGGPGVNYN
GVAISSSSFTNVGQDGKVQRGGGTKIITNRNGVIEEHTYPDDGSNINIVSSFPQPKIPKVPFLPLPPVPKIDPIV
IPKYRPGPNEEFRGRSFVSYRHSSNINGERSGGGVDTIINNDNGRVDKKTYKYGDLKDDF

PIENAPT00000019992, **PnSn1B** (*Pieris napi*)
MALRCVVAFLAVTCTHALNQYQNEYGNFQNNHGFRQPQFPSITMPPIKPIVFPTFPTFSPEDIINQRGGPGVNYN
GVAISSSSFTNVGQDGKVQRGGGTKIITNRNGVIEEHTYPDDGSNINIVLQFPQPKIPKFPVLPQIKLPLPLLPV
PPFPQIKVPFLPLPPVPKIDPIVIPKYRPGPNEEFRGRSFVSYRHSSNINGERSGGGVDTIINNDNGRVDKKTYK
YGDLKDDF

PIENAPT00000019991, **PnSn1C** (*Pieris napi*)
MPPIKPIVFPTFPTFSPEDIINQRGGPGVNYNGVAISSSSFTNVGQDGKVQRGGGTKIITNRNGVIEEHTYPDDG
SNINIVSSFPQPKIPKVPFLPLPPVPKIDPIVIPKYRPGPNEEFRGRSFVSYRHSSNINGERSGGGVDTIINNDN
GRVDKKTYKYGDLKDDF

PIENAPT00000008671, **PnSn2** (*Pieris napi*), partial sequence
YSVRITSGLPDPRGVGVYAYQDSLGNRYGGSYGLDDKDIVRNSDPYFGQYPTVAQVPYLGNFADSFYPNYISNYD
NILQEVFASNLEAQRLASYAAHKAYELTTNQIREFPNFSRFPSFGPIFGAGGLVPYDIMSRPNSAFASGAIGPGF
SHQIAAINPENDAMRNIEVTNRFNDAPGNNKFYGVSSSSYSSSSNVNGKAQNLRGAETVVNNNGKITKYRVQDP

PIENAPT00000007293, **PnSn3** (*Pieris napi*)

MIKIFVLFAAMCALVTGNTLSVEDFIKAASAGDFKTLRKFVNPNFAFDNFPGQPNLNTDVASLQPGPGAHVFGQA
ESSFSSYSNNNGKVSSESGGYGLINKDGVVSSYSFTPKNIVTPVENIPQN

maker-scaffold23-augustus-gene-1.98-mRNA-1, **PiSn1** (*Plodia interpunctella*)
MHTTNHFQHLHKNKHAYAHSAGYVWVDEEDDAPPGYKRMYVPPIMQPPPLPGFGNLPPLPQPPPLYVRDFSQPFE
HFHPFPQFVPINIPSPADIKNTKSEPGKVFNGVSVKSVSGFGEDEDGKPIRTGGTTIVVNENGEVKETKVGKNPP
NIED

maker-scaffold23-augustus-gene-1.96-mRNA-1, **PiSn2** (*Plodia interpunctella*)
MSCRKRTPVGVSSFAYQDSSGNRYGGTYHTPDGQLDQFFEPKDLQFQTPYQQNSYQAQGNFFPDYFRNLQFLIQD
ITETREFSRSGERRSNLTPLHPLRAIKPFLKPLPPLRPLRLFRPRHAKSGSGAFESNLANQALALKAAQQAFDLT
SSQAGYIPYLPFRSGDFGNFGNMFNSANSAFAGGFAGPGFTHQIAAISPPNPNNPNVNVFERFNEGSAIPGANFV
SMKSTAYSSSSNINGKEISNRGSETVVNNNGKVTGYSVHS

maker-scaffold23-exonerate_est2genome-gene-1.26-mRNA-1, **PiSn3** (*Plodia interpunctella*)
MTNFSVVVVLAAFVCALNANPTNFGSGGLDTSFDDAWEKLSISTPKPLTEQDIENLRPSNDTVVQGQSHYTSKHV
ETKNGVITNEESDTKDVTNDNGNVSKHETKN

BAS31058, **SrSn1A** (*Samia cynthia ricini*)
MAFTKILLALSFVALSNAFLLWPNGASSFAGSKNDYTGFPAPRFPLFPDFSGVLNFPTSFVSSAFSGFPRFPSVP
FPSPDDIRNTKARPGQTYTGLFTSSGGGGGGVIIANIDGQVLEKHFGETSSTTIDTLKKMAFTKILLALLSFVAL
S

BAS31061, **SrSn1B** (*Samia cynthia ricini*)
MAFTKILLALSFVALSNAFLLWPNGASSFAGSKMFPSFPSFGNLFNPFSILPPIINPDDIKTHVAGPNEHFSAVS
TSSHSFSSNVDGKVTEGGGMSTIVNDGKNVKESVLVYGDDDGDRDGDHDGNHDTDNDD

BAS31060, **SrSn1C** (*Samia cynthia ricini*)
MALMKIFLALLFVGLSNAVLMWPNDDPRFPSSKNYYTRYPAPGYPTFPDSSDFMNFPFKFVFNDFPRFQSFPKVP
FPSPDDIRNTNPRPGQTYTGLYASTGGSGGGVIIANVDGQVLEKRFPETTSDTSNN

GARL01003268 (translated to AA), **SeSn3A** (*Spodoptera exigua*)
MAKIVSTLILLIVIITVQGQAPEYEEVIKAARAGNWEQVQKILNSNLGKQAGWMPPSFDSLRELKPENGGHVYGE
AEYAYHSASNINGRTTEQSVGHKIINEDGRVKEFDFTPSTDYSPVIQSVISPIIQKAGDYGAAGYNQQYAGSGAG
KPNIAGQRLNNNLQQDYYPRNSANLALPVPHLSFTNVRDLKPPQGGHVYGEAQYAYHTASNINGKKSEDSGGHKI
INNDGKIKEFDFTPKPKFGPLKKLPLAVLDKDIGELKSLFLQ

GARL01003267 (translated to AA), **SeSn3A2** (*Spodoptera exigua*)
MAKIVSTLILLIVIITVQGQAPEYEEVIKAARAGNWEQVQKILNSNLGKQAGWMPPSFDSLRELKPENGGHVYGE
AEYAYHSASNINGRTTEQSVGHKIINEDGRVKEFDFTPSTDYSPVIQSVISPIIQKAGDYGAAGYNQQYAGSGAG
KPNIAGQRLNNNLQQDYYPRNSANLALPVPHLSFTNVRDLKPPQGGHVYGEAQYAYHTASNINGKKSEDSGGHKI
INNDGKIKEFDFTPKPKFGPVSYY

MG674523, **SliSn1A** (*Spodoptera littoralis*)
MGLSTFMLTAFLVVMTSGFNYGHFFLAGWPGWPTGDNPFAGYHSPMKYNFPPYKFKPIRPFKMKSVFDSFENFHN
FDDFPKFPEIKPIDFDMIKTYQPGNGENFQGHSIISNSYSSNNNGEISQGGHIAVVQNNNGEVKEEKIVF

MG674524, **SliSn1A2** (*Spodoptera littoralis*)
MGLSTFMLTAFLVVMTSGFNYGHFFLAGWPGWPTGDNPFAGYHSPMLSALRFGPISMPNPGFPKFPTIPSPTFLF
PFPTMPTFQSIQDMVKTMPNKGSYSGVVVSTQTESKMKDDGTLVKKGGSTIVINDDGKVTVHTSKFLLLN

MG674526, **SliSn1A4** (*Spodoptera littoralis*)
MGLSTFMLTAFLVVMTSAGWPGWPTGDNPFAGYHSPMLSALRFGPISMPNPGFPKFPTIPSPTFLFPFPTMPTFQ
SIQDMVKTMPNKGSYSGVVVSTQTESKMKDDGTLVKKGGSTIVINDDGKVTVHTSKFLLLN

MG674528, **SliSn2A** (*Spodoptera littoralis*)
MIKIIFLVMLPLAWSYGEPNLQGGGAFAYVDSSGHRYGGTYGLKDGRVIPTSGDPIPQRFADIAYPNEDDFGTAY
FANLENLLQEVFQRNIENQRLAFDAARKAFDLTSNQAGYYPTPNLASRFAFTNIPSLPLPMPMPHFGSNSAFAGA
AAGPGFQHQVASIFPVNPANPNVNHFADTVQQPGRNGFYSVSSNSFSSSSNVNGKTLNSRGAETLVNDNGRITHY
KVEN

MG674529, **SliSn2B** (*Spodoptera littoralis*)
MIKIIFLVMLPLAWSYGEPNLQGGGAFAYVDSSGHRYGGTYGLKDGRVIPTSGDPIPQRFADIAYPNEDDFGTAY
FANLENLLQEFAFTNIPSLPLPMPMPHFGSNSAFAGAAAGPGFQHQVASIFPVNPANPNVNHFADTVQQPGRNGF
YSVSSNSFSSSSNVNGKTLNSRGAETLVNDNGRITHYKVEN

EZ983678 (translated to AA), **SliSn3** (*Spodoptera littoralis*)
MARIISTLILVVVVIAVQGQAPEYEEVIKAAKAGNWDQVQELLNSNLGKQAGWKPPSFNSLRELKPENGGHVYGE
AEYAYHSASNVNGRTTEQNAGHKIINEDGRVKEYDFTPSTSYAPHIPSIISPVGQKAGDYVASDYHYQYGGSVSG
KPNFASQKLNNNLPQDYYPRNSAVVPIPALSFTNVRELKPPQGGHVYGEAQYAYHTASNINGQKSEDAGGHKIIN
NDGKVKEFDFTPKPISGPLKKLPLAVLDKNIGRVKSLILQ

XM_022965570 (translated to AA), **SlSn1** (*Spodoptera litura*)
MGLSTFMLTAFLVVMTSGFNYGHFFLAGWPGWPTGDNPFAGYHSPMLTSLRFGPISMPNPGIPKFPTIPSPTFLF
PFPTMPTFQSIQDMVKTMPNKGSYSGVVVSTQTESKMKDDGTLVKKGGSTILINDDGKVTVHTTGNNPPDLNQIK
PTTITLHQQKYNFPPYKFRPIRPFKLKNVFDSFENFQNFDDFPKFPEIKPIDFDIIKTYQPGNGENFRGHSIISN
SYSTNNNGQVSQGGHIAVVQNDNGQVKEEKIVF

XM_022966172 (translated to AA), **SlSn2A** (*Spodoptera litura*)
MIKIIFLVMLPLAWSYGEPNLQGGGAFAYVDSSGHRYGGTYGLKDGRVIPTSGDPIPQRFADFAYPNEDDFSTAY
FANLENLLQEVFQRNIENQRLAFDAARKALDLTSNQAGYYPTPNLASRFAFTNIPSLPLPMPMPHFGSNSAFAGA
AAGPGFQHQVASIFPVNPANPNVNHFADTVQQPGRNGFYSVSSNSFSSSSNVNGKAVNSRGAETLVNDNGRITHY
KVEN

XM_022966173 (translated to AA), **SlSn2B** (*Spodoptera litura*)
MIKIIFLVMLPLAWSYGEPNLQGGGAFAYVDSSGHRYGGTYGLKDGRVIPTSGDPIPQRFADFAYPNEDDFSTAY
FANLENLLQEFAFTNIPSLPLPMPMPHFGSNSAFAGAAAGPGFQHQVASIFPVNPANPNVNHFADTVQQPGRNGF
YSVSSNSFSSSSNVNGKAVNSRGAETLVNDNGRITHYKVEN

XP_022822185, **SlSn3** (*Spodoptera litura*)
MARIISTLILVVVVIAVQGQAPEYEEVIKAAKAGNWDQVQELLSNLGKQAGWKPPSFNSLRELKPENGGHVYGEA
EYAYHSASNVNGRTTEQNAGHKIINEDGRVKEYDFTPSTSYAPHIPSIISPVGQKAGDYVASDYHYQYGGSVSGK
PNFASQKLNNNLPQDYYPRNSAVVPIPALSFTNVRELKPPQGGHVYGEAQYAYHTASNINGQKSEDAGGHKIINN
DGKVKEFDFTPKPISGPLKKLPLAVLDK

GBAS01010279 (translated to AA), **TlSn1** (*Telchin licus*)
MALKKIIFITVSLAAMVHAGYVWQDDDQDMWPRYQAYNMPNPYFPPLPALPPLPSLPTFGHFSFPPFPPLPRFPP
LPKIPTPGEISKIKPAPGQVVSGIAVKSSSGIATDKDGNVIHSGGTTVVTNDNGDVKEIKIGKSPPNINDPIIIP
PFPEFKFEPIPPIPPIPPIDFDDIRSYQPDPDENFVAYSTSSHSHSSNINGKKSSGGGTSTILNVNGAVDEEAVR
IGQHDKKKKKKKY

MG674530, **TbSn1** (*Tineola bisselliella*)
MIIKLFLLLTTFVVISQADMDFPPFPEFPKMHFPKMEFRPLRFPKIVIPSPLEMRQMKPRPGETFHGVAVQSVSG
SEVDESGKVVIKEGATVITNHNGNVKEFRYDHPDESPVVEFAVSRK

MG674531, **TbSn3** (*Tineola bisselliella*)
MAKFTLLFVIATLAVCVQALPYRQNDVASMAVQQKWEELSRLLNERIQARFGLTPVDPNVFQGRHFDIKNLHPTD
GGHVYGESKFEYHTATNVNGHEDKHSGGHKVINKDGHVTEFDYTPQN

Supplementary figure legends

Figure S1. Alignments of amino acid sequences of the Sn1 seroins. Line at the bottom of the figure indicates range of individual modules. Fuzzy borders of the modules are indicated by the broken line. Note that 18 top sequences represent the N-version, the bottom 17 sequences the C-versions, and the remainder are the L-versions of Sn1 seroins.

Figure S2. Alignments of amino acid sequences of the Sn2 seroins. Line at the bottom indicates range of individual modules. Fuzzy borders of the modules are indicated by the broken line. The sequences from B1 to C2 are highly diversified due to alternative splicing.

Figure S3. Alignment of amino acid sequences of the Sn3 seroins. The segments of the bottom line indicate the fuzzy borders of the modules. The large modules B2 and C2 were found in most noctuids but in no species from other superfamilies.

Figure S4. Alignment of 117 seroin protein sequences used for the construction of phylogenetic trees (shown in Figures 3 and S5).

Figure S5. Phylogenetic trees based on 117 seroin protein sequences. (**A)** Maximum likelihood tree reconstructed with NNI search algorithm, statistical evaluation calculated by aBayes test is shown next to the branches, only values higher than 50 are presented. (**B)** Maximum likelihood tree reconstructed with SPR search algorithm (from 10 random trees), statistical evaluation calculated by aBayes test is shown next to the branches, only values higher than 50 are presented. (**C)** Bayesian inference tree based on 3,000,000 generations (50% burn-in), nodes support values are Bayesian posterior probabilities, only values higher than 0.95 are shown.

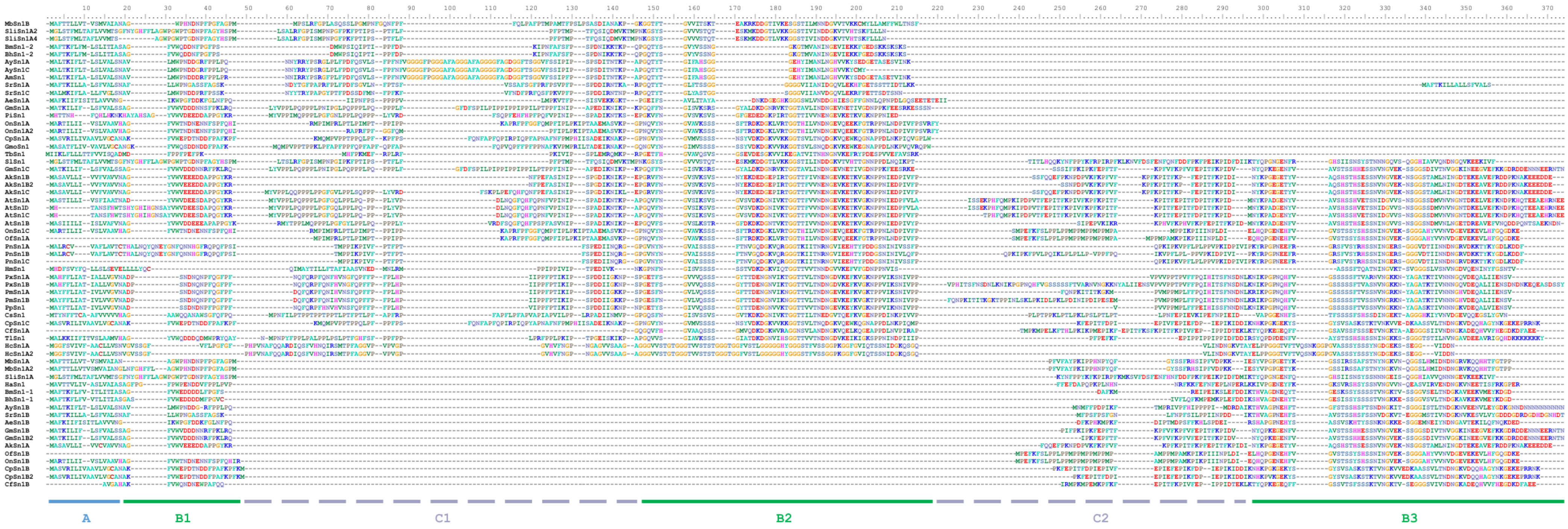

Fig. S1

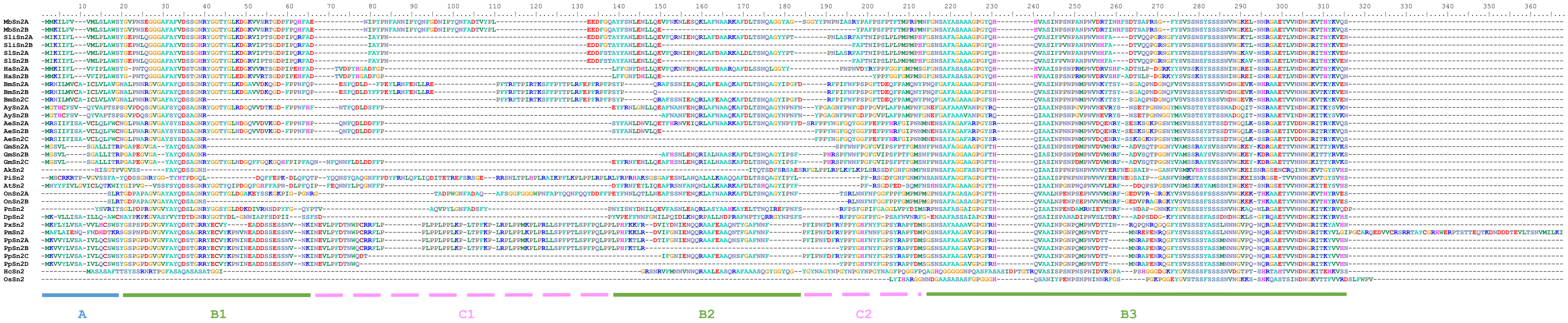
MbSn2A
MbSn2B
SliSn2A
SliSn2B
SlSn2A
SlSn2B
HaSn2A
HaSn2B
BmSn2A
BmSn2B
BmSn2C
AySn2A
AySn2B
AeSn2A
AeSn2B
AeSn2C
GmSn2A
GmSn2B
GmSn2C
AkSn2
PiSn2
AtSn2
OnSn2A
OnSn2B
PnSn2
DpSn2
PxSn2
PmSn2
PpSn2A
PpSn2B
PpSn2C
PpSn2D
HcSn2
OsSn2
A
B1
C1
B2
C2
B3

Fig. S2

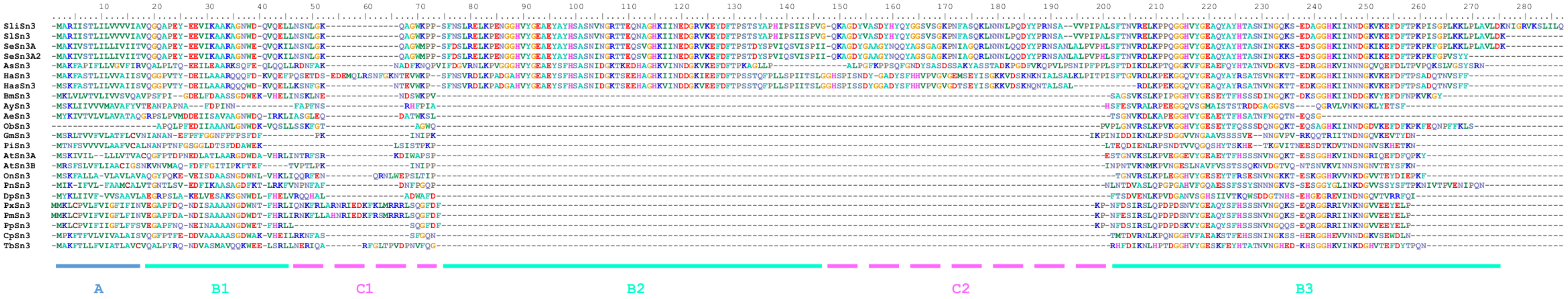
SliSn3
SlSn3
SeSn3A
SeSn3A2
AsSn3
HaSn3
HasSn3
BmSn3
AySn3
AeSn3
ObSn3
GmSn3
PiSn3
AtSn3A
AtSn3B
OnSn3
PnSn3
DpSn3
PxSn3
PmSn3
PpSn3
CpSn3
TbSn3
A
B1
C1
B2
C2
B3

Fig. S3

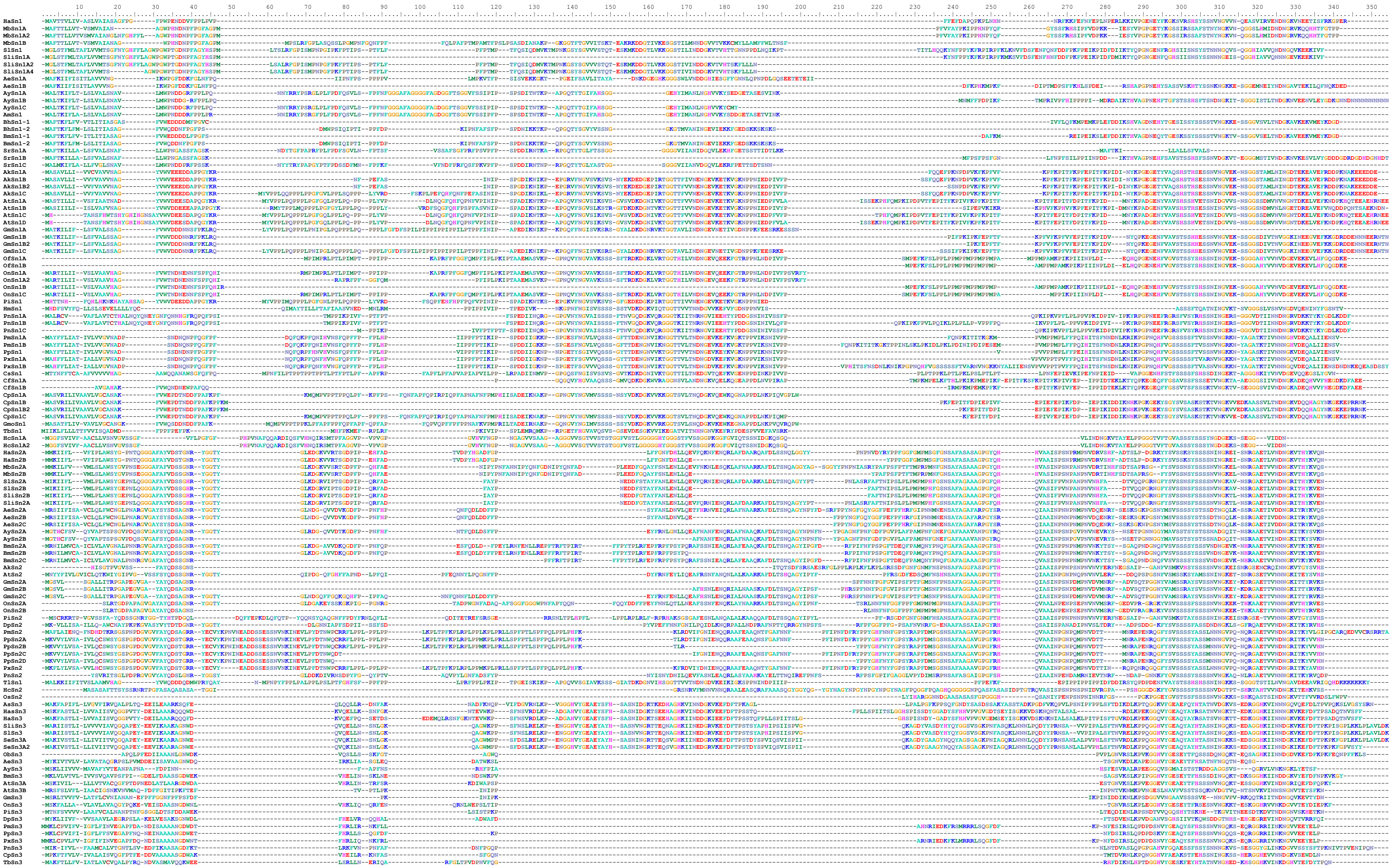

Fig. S4

Fig. S5A

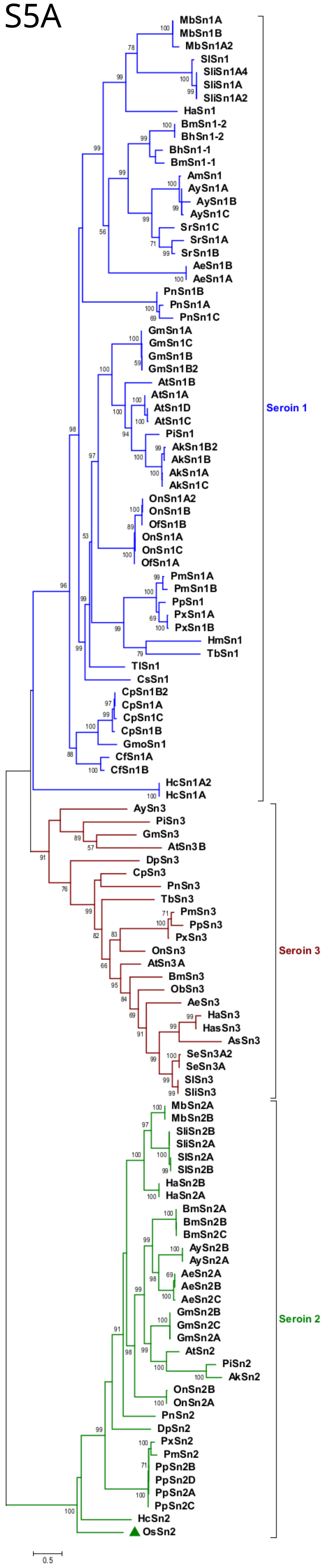
MbSn1A
MbSn1B
MbSn1A2
SlSn1
SliSn1A4
SliSn1A
SliSn1A2
HaSn1
BmSn1-2
BhSn1-2
BhSn1-1
BmSn1-1
AmSn1
AySn1A
AySn1B
AySn1C
SrSn1C
SrSn1A
SrSn1B
AeSn1B
AeSn1A
PnSn1B
PnSn1A
PnSn1C
GmSn1A
GmSn1C
GmSn1B
GmSn1B2
AtSn1B
AtSn1A
AtSn1D
AtSn1C
PiSn1
AkSn1B2
AkSn1B
AkSn1A
AkSn1C
OnSn1A2
OnSn1B
OfSn1B
OnSn1A
OnSn1C
OfSn1A
PmSn1A
PmSn1B
PpSn1
PxSn1A
PxSn1B
HmSn1
TbSn1
TlSn1
CsSn1
CpSn1B2
CpSn1A
CpSn1C
CpSn1B
GmoSn1
CfSn1A
CfSn1B
HcSn1A2
HcSn1A
Seroin 1
AySn3
PiSn3
GmSn3
AtSn3B
DpSn3
CpSn3
PnSn3
TbSn3
PmSn3
PpSn3
PxSn3
OnSn3
AtSn3A
BmSn3
ObSn3
AeSn3
HaSn3
HasSn3
AsSn3
SeSn3A2
SeSn3A
SlSn3
SliSn3
Seroin 3
MbSn2A
MbSn2B
SliSn2B
SliSn2A
SlSn2A
SlSn2B
HaSn2B
HaSn2A
BmSn2A
BmSn2B
BmSn2C
AySn2B
AySn2A
AeSn2A
AeSn2B
AeSn2C
GmSn2B
GmSn2C
GmSn2A
AtSn2
PiSn2
AkSn2
OnSn2B
OnSn2A
PnSn2
DpSn2
PxSn2
PmSn2
PpSn2B
PpSn2D
PpSn2A
PpSn2C
HcSn2
OsSn2
Seroin 2
0.5

Fig. S5B

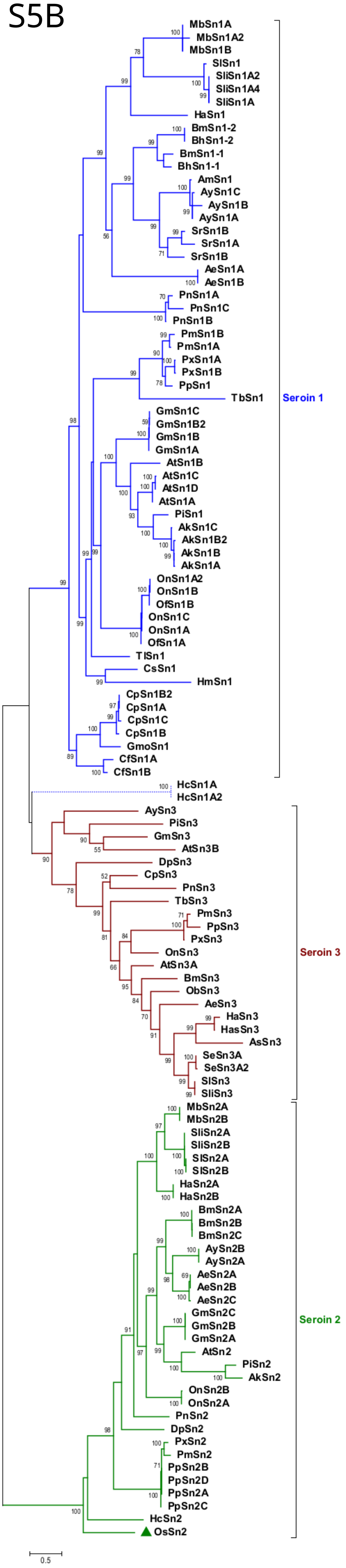

MbSn1A
MbSn1A2
MbSn1B
SlSn1
SliSn1A2
SliSn1A4
SliSn1A
HaSn1
BmSn1-2
BhSn1-2
BmSn1-1
BhSn1-1
AmSn1
AySn1C
AySn1B
AySn1A
SrSn1B
SrSn1A
SrSn1B
AeSn1A
AeSn1B
PnSn1A
PnSn1C
PnSn1B
PmSn1B
PmSn1A
PxSn1A
PxSn1B
PpSn1
TbSn1
Seroin 1
GmSn1C
GmSn1B2
GmSn1B
GmSn1A
AtSn1B
AtSn1C
AtSn1D
AtSn1A
PiSn1
AkSn1C
AkSn1B2
AkSn1B
AkSn1A
OnSn1A2
OnSn1B
OfSn1B
OnSn1C
OnSn1A
OfSn1A
TlSn1
CsSn1
HmSn1
CpSn1B2
CpSn1A
CpSn1C
CpSn1B
GmoSn1
CfSn1A
CfSn1B
HcSn1A
HcSn1A2
AySn3
PiSn3
GmSn3
AtSn3B
DpSn3
CpSn3
PnSn3
TbSn3
PmSn3
PpSn3
PxSn3
OnSn3
AtSn3A
BmSn3
ObSn3
AeSn3
HaSn3
HasSn3
AsSn3
SeSn3A
SeSn3A2
SlSn3
SliSn3
Seroin 3
MbSn2A
MbSn2B
SliSn2A
SliSn2B
SlSn2A
SlSn2B
HaSn2A
HaSn2B
BmSn2A
BmSn2B
BmSn2C
AySn2B
AySn2A
AeSn2A
AeSn2B
AeSn2C
GmSn2C
GmSn2B
GmSn2A
AtSn2
PiSn2
AkSn2
OnSn2B
OnSn2A
PnSn2
DpSn2
PxSn2
PmSn2
PpSn2B
PpSn2D
PpSn2A
PpSn2C
HcSn2
OsSn2
Seroin 2
0.5

Fig. S5C

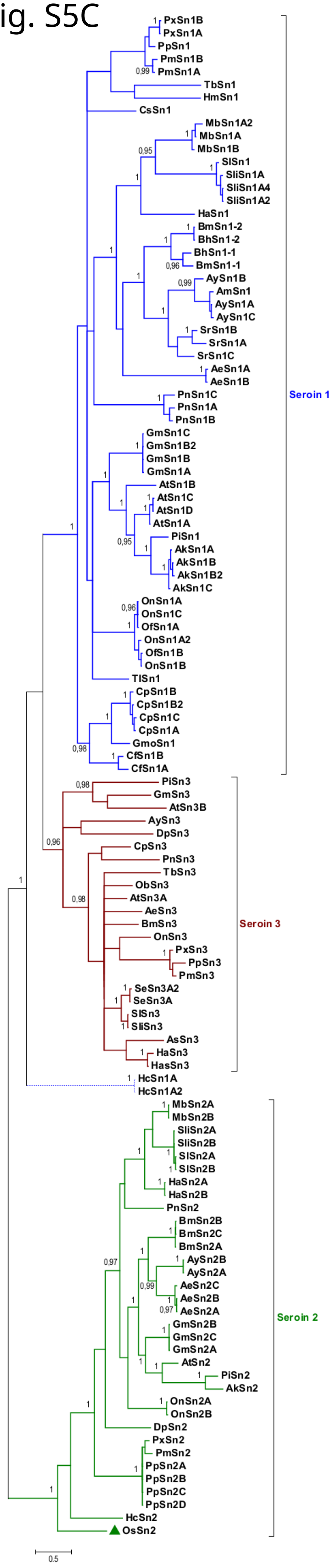

PxSn1B
PxSn1A
PpSn1
PmSn1B
PmSn1A
TbSn1
HmSn1
CsSn1
MbSn1A2
MbSn1A
MbSn1B
SlSn1
SliSn1A
SliSn1A4
SliSn1A2
HaSn1
BmSn1-2
BhSn1-2
BhSn1-1
BmSn1-1
AySn1B
AmSn1
AySn1A
AySn1C
SrSn1B
SrSn1A
SrSn1C
AeSn1A
AeSn1B
PnSn1C
PnSn1A
PnSn1B
GmSn1C
GmSn1B2
GmSn1B
GmSn1A
AtSn1B
AtSn1C
AtSn1D
AtSn1A
PiSn1
AkSn1A
AkSn1B
AkSn1B2
AkSn1C
OnSn1A
OnSn1C
OfSn1A
OnSn1A2
OfSn1B
OnSn1B
TlSn1
CpSn1B
CpSn1B2
CpSn1C
CpSn1A
GmoSn1
CfSn1B
CfSn1A
Seroin 1
PiSn3
GmSn3
AtSn3B
AySn3
DpSn3
CpSn3
PnSn3
TbSn3
ObSn3
AtSn3A
AeSn3
BmSn3
OnSn3
PxSn3
PpSn3
PmSn3
SeSn3A2
SeSn3A
SlSn3
SliSn3
AsSn3
HaSn3
HasSn3
Seroin 3
HcSn1A
HcSn1A2
MbSn2A
MbSn2B
SliSn2A
SliSn2B
SlSn2A
SlSn2B
HaSn2A
HaSn2B
PnSn2
BmSn2B
BmSn2C
BmSn2A
AySn2B
AySn2A
AeSn2C
AeSn2B
AeSn2A
GmSn2B
GmSn2C
GmSn2A
AtSn2
PiSn2
AkSn2
OnSn2A
OnSn2B
DpSn2
PxSn2
PmSn2
PpSn2A
PpSn2B
PpSn2C
PpSn2D
HcSn2
OsSn2
Seroin 2
0.5
